# Pseudo-spectral angle mapping for pixel and cell classification in highly multiplexed immunofluorescence images

**DOI:** 10.1117/1.JMI.11.6.067502

**Published:** 2024-12-10

**Authors:** Madeleine S. Torcasso, Junting Ai, Gabriel Casella, Thao Cao, Anthony Chang, Ariel Halper-Stromberg, Bana Jabri, Marcus R. Clark, Maryellen L. Giger

**Affiliations:** a The University of Chicago, Department of Radiology, Chicago, Illinois, United States; b The University of Chicago, Department of Medicine, Section on Rheumatology, Chicago, Illinois, United States; c The University of Chicago, Pritzker School of Molecular Engineering, Chicago, Illinois, United States; d The University of Chicago, Department of Pathology, Chicago, Illinois, United States; e The University of Chicago, Department of Medicine, Section on Gastroenterology, Hepatology and Nutrition, Chicago, Illinois, United States

**Keywords:** image analysis, cell classification, highly multiplexed imaging, microscopy, cellular imaging

## Abstract

**Purpose:**

The rapid development of highly multiplexed microscopy has enabled the study of cells embedded within their native tissue. The rich spatial data provided by these techniques have yielded exciting insights into the spatial features of human disease. However, computational methods for analyzing these high-content images are still emerging; there is a need for more robust and generalizable tools for evaluating the cellular constituents and stroma captured by high-plex imaging. To address this need, we have adapted spectral angle mapping—an algorithm developed for hyperspectral image analysis—to compress the channel dimension of high-plex immunofluorescence (IF) images.

**Approach:**

Here, we present pseudo-spectral angle mapping (pSAM), a robust and flexible method for determining the most likely class of each pixel in a high-plex image. The class maps calculated through pSAM yield pixel classifications which can be combined with instance segmentation algorithms to classify individual cells.

**Results:**

In a dataset of colon biopsies imaged with a 13-plex staining panel, 16 pSAM class maps were computed to generate pixel classifications. Instance segmentations of cells with Cellpose2.0 (F1-score of 0.83±0.13) were combined with these class maps to provide cell class predictions for 13 cell classes. In addition, in a separate unseen dataset of kidney biopsies imaged with a 44-plex staining panel, pSAM plus Cellpose2.0 (F1-score of 0.86±0.11) detected a diverse set of 38 classes of structural and immune cells.

**Conclusions:**

In summary, pSAM is a powerful and generalizable tool for evaluating high-plex IF image data and classifying cells in these high-dimensional images.

## 1 Introduction

The recent emergence of highly multiplexed tissue imaging modalities—systems that go beyond the traditional channel limit of immunofluorescence (IF) microscopy—has enabled the study of cells while maintaining spatial context, which is integral for furthering our understanding of immunity in human disease.^1^^–^^7^ Studying cells within native tissue is imperative for elucidating the organizational principles of cells and studying cell:cell interactions that may otherwise be disrupted.^6^^–^^10^ Tissue-destructive methods such as single-cell sequencing can provide specific information about gene expression in cells,^11^^–^^13^ but such methods lack the spatial context provided by imaging and are therefore constrained in the information they can convey. Highly multiplexed imaging addresses the need for higher specificity in studying a variety of cell phenotypes in their native environment; multiple methodologies have been developed to image upward of 40 protein markers in one field of view.^1^^,^^5^^,^^14^ Although these imaging methods demonstrate the potential to unlock new insights into cell–cell interactions *in situ*, there is still a need for robust and reliable methods for quantifying these types of images.

Most existing methods for characterizing high-plex IF images perform object detection and/or instance segmentation of cells first, followed by annotation of cells.^7^^,^^15^ Typically, detected cells are classified using their average pixel value across the segmentation mask, or the mean pixel intensity (MPI), from each channel.^15^^–^^17^ However, averaging intensity across all image pixels that fall within a cell mask loses potentially valuable information provided by signal localization within a cell. In addition, IF imaging captures only the two-dimensional section of cells and structures existing in a three-dimensional space. Many cells, particularly some populations of immune cells, can have protrusions that reach in and out of the imaging plane, potentially resulting in detected signals not being assignable to a cell nucleus in the imaging plane, or interfering with the classification of in-plane cells.^9^^,^^18^^,^^19^ Detection and characterization of cells in the imaging plane will provide integral information for spatial biology studies; however, the spatial context of the tissue surrounding cells is also worth understanding. We propose methods that can retain valuable information from the interstitial spaces between cells while providing an automated framework for rapid annotation of known cell types by collapsing the “spectral” or “channel” dimension of highly multiplexed IF images.

**Fig. 1:**
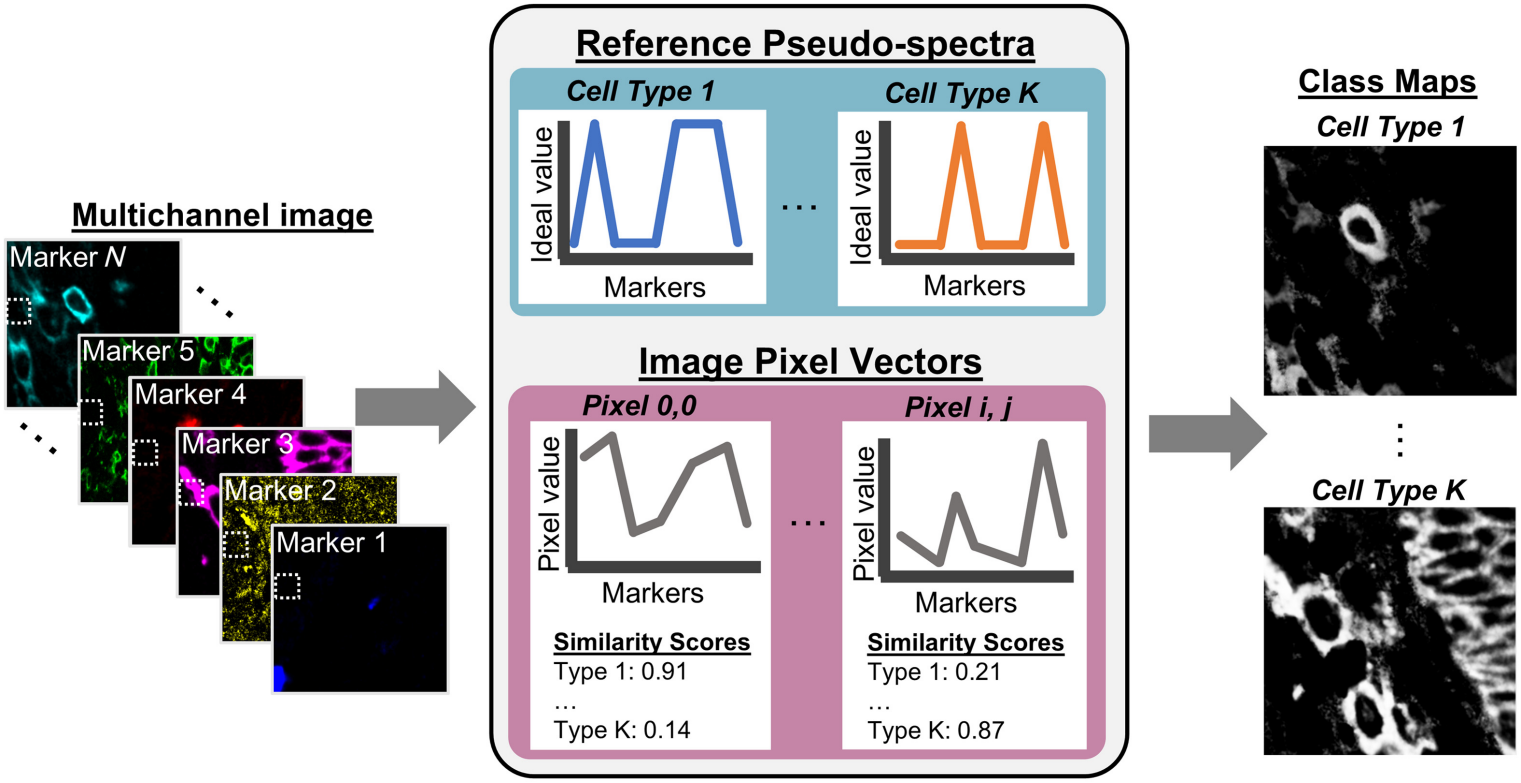
Pseudo-spectral angle mapping in highly multiplexed immunofluorescence images classifies pixels by their cosine similarity to ideal reference profiles marker expression.

Annotation of cell classes with respect to cell type and cell state in images is a particularly difficult computational task.^9^^,^^15^^,^^20^ The boundary between cell class and cell state can be subjective, and cell class ground truth is both noisy and difficult to acquire; human readers are not reliable in defining ground truth for cells in high-channel images.^21^ Because of the scarcity of reliable ground truth and training data, methods such as support vector machines or convolutional neural networks tend to perform poorly. Other methods classify cells by their protein expression (represented by MPI) across several markers, either through a decision tree-based method or through clustering on cell features, usually MPI of each channel.^15^ Cell typing in high-plex images is specific to the staining panel used in each imaging experiment, which means models trained with specific image channels or with channel MPI as input features are typically not generalizable to new datasets. For example, an imaging experiment that does not include blood dendritic cell antigen 2 (BDCA2) in the staining panel cannot directly probe plasmacytoid dendritic cells (pDCs), and models trained with this data would therefore lack the ability to differentiate pDCs from other dendritic cells (DCs). Because of the variety of markers required to identify specific immune cell types and states, there is a need to develop more accessible, flexible, and reliable methods for studying spatial immunology. Here, we present an adaptation of a spectral angle mapping—a concept drawn from hyperspectral image analysis^22^^–^^24^—to calculate pixel-level class representations for cell class annotation on highly multiplexed IF imaging data (Fig. 1).

Although high-plex IF imaging does not physically collect hyperspectral data, a single-channel image of protein expression can be likened to a single wavelength band of a hyperspectral image. As this dimension of high-plex IF data is not truly spectral, we refer to data in the channel dimension of our images as “pseudo-spectral.” Pseudo-spectral angle mapping (pSAM) compresses the channel dimension of high-plex images into class maps through vector similarity calculations. Reference vectors used for these vector similarity calculations can be generated for any arbitrary staining panel, making pSAM more generalizable than current techniques, specifically across imaging experiments using different staining panels. We demonstrate that pSAM is a readily adaptable method that will expedite the quantification of highly multiplexed imaging data for studying spatial immunity and spatial biology.

## 2 Related Work

### 2.1 Cell Classification in High-Plex IF Images

High-plex IF imaging is a powerful tool for investigating cells *in situ*, but characterizing cells in high-plex image data comes with challenges. Unlike single-cell experiments, classifying and characterizing cells in tissue can be affected by tissue autofluorescence, true signal from neighboring cells, inaccurate cell segmentation, image pre-processing artifacts, and batch effects from staining and imaging.^15^^,^^25^^,^^26^ Most existing methods for cell annotation in high-plex image data perform cell localization and segmentation first then characterize the detected cells by evaluating the pixel intensities within each cell mask across all relevant image channels.^15^ One popular method for cell annotation is reminiscent of flow cytometry analysis: applying manual or semi-automatic gates to MPI across the population or detected cells, following decision-tree logic until all cells have reached a terminal branch.^14^^,^^27^ Clustering algorithms are also commonly used to separate cells into specific phenotypes.^17^^,^^28^

These decision tree and clustering algorithms are dataset-specific and typically not generalizable to new data. Decision trees are known to be unstable, as small changes in the data distribution can produce drastically different results.^29^ Even on the same data with the same normalization, slightly different manual gates affect downstream decision points and cell classifications. Practically, these methods are typically iterative, requiring multiple rounds of human intervention. Brbić et. al.^30^ proposed a graph-based deep learning method that can potentially generalize to new datasets and minimize the need for human intervention.^17^ This graph-based method also includes cell location to improve classification. All of these “cell-first” methods for cell classification share limitations related to batch effects and signal overlap from neighboring cells. Some algorithms, such as REDSEA,^25^ have been developed to try to mitigate some of these limitations. Normalization and batch effect correction are also integral steps for ensuring accurate classification with these methods.^15^

### 2.2 Spectral Angle Mapping and Pixel Classification

Hyperspectral imaging, originally known as imaging spectroscopy, is a decades-old imaging technique developed for remote sensing.^31^ Spectral angle mapping (SAM) was first applied for hyperspectral image and pixel classification and clustering in 1993.^24^ To achieve accurate classifications of pixels in remote sensing data, a cosine similarity metric [Eq. (1)] was used to compute the angular distances between a detected spectrum (**A**) and spectra of known ground surface materials (**B**). As the advent of this pixel classification scheme, SAM and its variants have been used for pixel classification in remote sensing images and novel applications of hyperspectral imaging, such as label-free imaging of biospecimens^22^^,^^32^^–^^35^

$$\cos(\theta )=\frac{A\cdot B}{\left\|A\|\|B\right\|}.$$ (1)

Clear parallels can be drawn between hyperspectral image data and high-plex IF data. Rather than wavelength along the channel dimension of the image, high-plex IF has protein-specific markers. Pixel vectors from a high-plex IF image do not contain spectra in the physical sense, but the variable intensity across channels can be interpreted as such. Lui et al. developed Pixie,^1^ a method for pixel-level annotation (classification) in high-plex IF images. Pixie relies on clustering for pixel classification. However, due to the immense number of pixels in a whole-slide image, current hardware limitations require sampling of pixels, and therefore, Pixie is only trained on a small fraction of pixel vectors from the image. This potentially limits the ability of the algorithm to detect rare classes of pixels.

pSAM leverages the interpretability and simplicity of spectral angle-based pixel classification methods as a novel approach to solving the cell classification problems presented by high-plex IF imaging. This method allows researchers to incorporate known biology into cell classification while mitigating many of the challenges that come from a “cell-first” classification method. These “cell-first” approaches prioritize reducing the image into objects (cells) then extract features of those cells for classification. Conversely, pSAM takes a “class-first” approach, maintaining full spatial resolution but compressing the channel dimension of the image into class maps prior to pulling cells out of the data. This “class-first” approach overcomes many previously mentioned limitations of the “cell-first” approach, including batch effects and the spatial bleed through of signal into neighboring cell segmentations.

## 3 Methods

### 3.1 Sample and Image Acquisition

Thirteen formalin-fixed, paraffin-embedded (FFPE) colon biopsy samples from patients diagnosed with primary sclerosing cholangitis (PSC) and/or inflammatory bowel disease (IBD) were acquired from the Human Tissue Resource Center at the University of Chicago (HTRC). In addition, three kidney samples were selected from existing datasets also acquired from the HTRC. One kidney sample was from a patient diagnosed with lupus nephritis (LuN), one from a patient with mixed rejection of renal allograft [referred to later as kidney transplant rejection (KTR)], and one from a patient with angiomyolipoma. All kidney samples were also FFPE.

A 5-μm section of each biopsy was de-paraffinized and mounted on a functionalized coverslip for iterative staining and imaging. Colon samples were iteratively stained and imaged with a 13-marker panel (Table S1 in the Supplementary Material), and kidney samples were iteratively stained and imaged with a 43-marker panel (Table S2 in the Supplementary Material), which included 10/13 markers from the colon panel. The PhenoCycler protocol was used for iterative staining.^36^ All samples were imaged on an Andor Dragonfly spinning disk confocal microscope with a 40× objective lens. For the colon dataset, each cycle of imaging included a nucleus stain [4′,6-diamidino-2-phenylindole (DAPI)] imaged at 405 nm and three other stains from the panel imaged at 488, 561, and 637 nm. For the kidney samples, each imaging cycle contained DAPI and four other stains, with an additional channel at 730 nm. Blank imaging cycles (no fluorescence reporters except for DAPI) were acquired before and after all staining cycles to capture background tissue autofluorescence. The resulting full-section images had a 0.1507-μm pixel size.

### 3.2 Image Preprocessing

Images were acquired in 2048×2048  pixel fields of view with a 205-pixel (∼10%) overlap in each dimension with neighboring tiles. The Ashlar stitching and alignment software^37^ was used to stitch all image tiles into full-section composites and align all imaging cycles to the DAPI channel from the first staining cycle.^37^ Ashlar performance was visually checked across all samples. After aligning all image channels, the first blank cycle of imaging was used for background subtraction and spectral normalization of all stained images. First, each channel of the blank cycle was subtracted from the corresponding fluorescence channel in all imaging cycles. Each imaging wavelength has a different dynamic range, so the subtracted images were also divided by the standard deviation of the background image to standardize dynamic range across imaging wavelengths. After standardization relative to imaging wavelength, images were min–max normalized to the 99th percentile.

### 3.3 Defining Reference Pseudo-Spectra

**Fig. 2:**
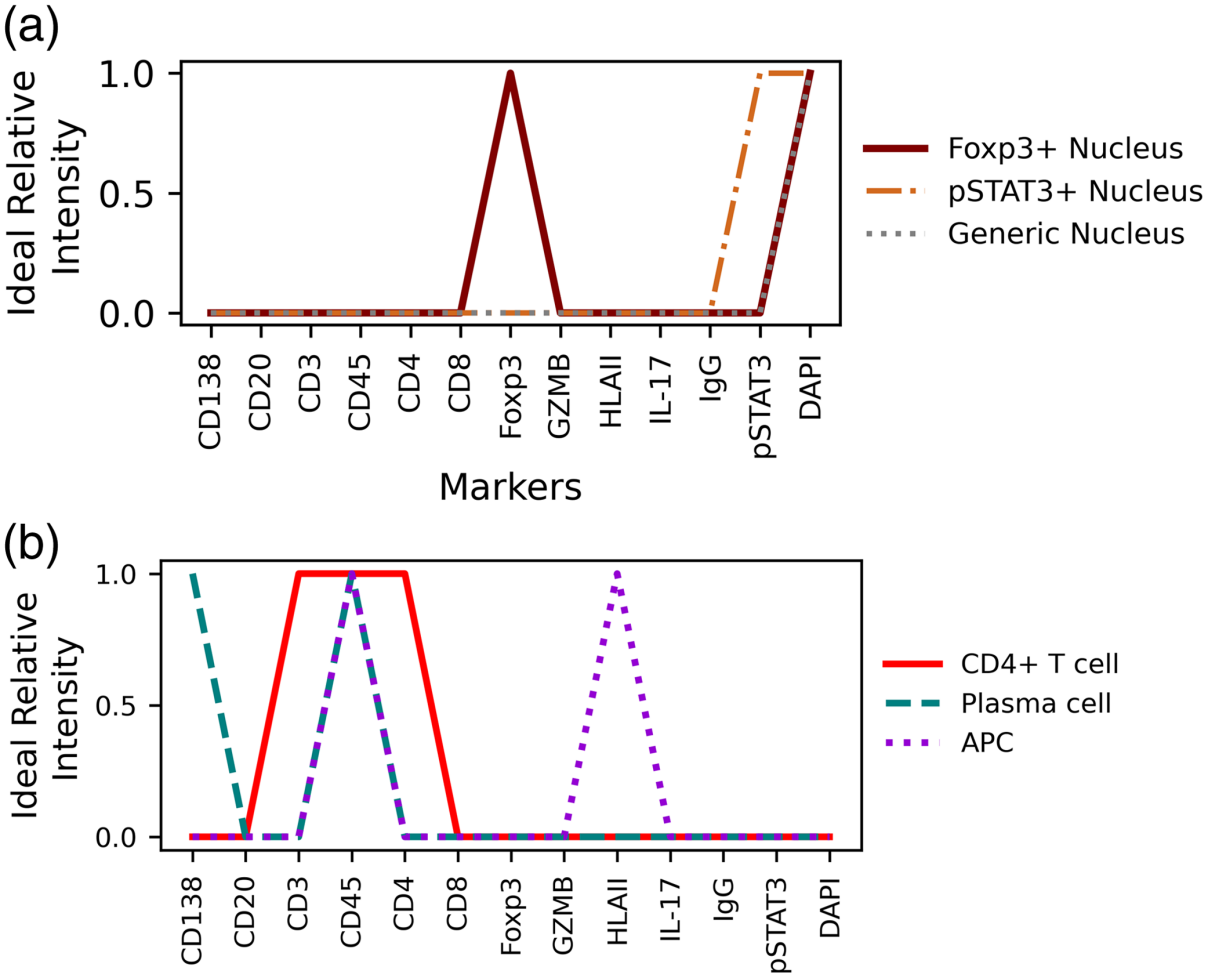
Reference pseudo-spectra for the PSC dataset were designed for specific cell compartments such as the nucleus (a) or cell membrane (b), based on the molecular target of the marker.

The specific reference pseudo-spectra for each panel were generated through a literature review and with expert immunologist input to reflect the protein expression patterns for each cell class. Because certain markers are known to localize to different compartments of the cell (i.e., nucleus, cytoplasm, or membrane), compartment-specific references were defined (Fig. 2). For the PSC dataset, reference (or “ideal”) pseudo-spectra were defined with binary values (0 or 1) for each marker (Fig. S1 in the Supplementary Material). In more complex panels such as the kidney panel, there are situations in which a marker is expected to be expressed at multiple levels (high/low/no expression). In these cases, a value of 0.5 was used to capture low expression (Fig. S2 in the Supplementary Material). Importantly, marker order was held consistent across all defined references. After visual quality checks of the images, some references were adapted, and new references were generated due to the cross-reactivity of some markers. The “tubule nucleus” reference spectra used for the kidney data defined CD21 as high in the nucleus compartment of several tubule cells in these pathological states.

Given the wealth of literature defining immune cells based on their gene or protein expression,^38^^–^^41^ and the help of expert immunologists, we generated ideal pseudo-spectra as models of the cell types probed by two high-plex staining panels. For example, an exhausted CD8 T cell will have high expression of the markers CD45, CD3, CD8, and PD1 on the surface of the cell, whereas a T regulatory cell will have high expression of CD45, CD3, and CD4 on the surface of the cell and high expression of Foxp3 in the nucleus of the cell. In addition, images were examined for cross-reactivity of the antibodies. In the kidney dataset, CD21 was selected to identify germinal center B cells. However, this marker showed a notably high signal in many tubule cell nuclei of the kidney. Therefore, we also generated a reference pseudo-spectrum for a “tubule nucleus” class, even though an antibody specific to tubule cell nuclei was not used in the staining panel.

In the 13-marker panel used for staining colon biopsies from PSC and IBD patients, three markers probed cell nuclei, resulting in three unique ideal pseudo-spectra for the nucleus compartment [Fig. 2(a)]. The remaining 10 markers are expected to be expressed on the cell membrane or within the cytoplasm of cells,^42^^–^^44^ collectively referred to here as the membrane compartment. Three of the 13 reference pseudo-spectra for this compartment are shown in Fig. 2(b), with all reference profiles displayed in Fig. S1 in the Supplementary Material. In total, 16 unique reference pseudo-spectra were generated from the 13-marker panel used in the PSC imaging experiment. Notably, there are more cell classes that could exist in these inflamed colon samples which cannot be captured by this 13-marker staining panel.

The kidney dataset contained 43 markers, 30 of which were used to define reference pseudo-spectra. Thirty-eight unique references were generated from these 30 markers: 34 membrane-associated pseudo-spectra and 4 nucleus-associated pseudo-spectra (Fig. S2 in the Supplementary Material). For the kidney dataset, we also expect a contingent of unclassified cells: some that are not captured by the staining panel and some that are not captured by this set of references. We generated 38 total references from this panel, but more references could be generated from these 43 markers that would further sub-type some of our selected classes.

### 3.4 Pseudo-Spectral Angle Mapping

All image channels were consistently stacked in the order defined by the reference pseudo-spectra. Each channel of the multi-channel stack was normalized to match the range of the reference pseudo-spectra (0 to 1). Pixel vectors extracted from the image data were defined as the value of all markers at a single (x,y) position. Each pixel vector A in the image was compared with all reference pseudo-spectra B by computing the cosine similarity between the two vectors [Eq. (1)]. After all vector similarity calculations were complete, class maps for all references were stacked. Pixel class was defined as the argmax of the class map stack (Θ) at each location [Eq. (2)]

$${\mathrm{class}}_{i,j}=\arg\;\underset{x}{\max}(\Theta_{i,j}(x)).$$ (2)

### 3.5 Instance Segmentation of Cells

The DAPI channel of each image was used to generate instance segmentations of all cells in each full-section image. The “nuclei” model in Cellpose2.0^45^ was fine-tuned using human-in-the-loop training for each dataset. The three kidney samples used for the generalization experiments originated from separate datasets. Separate models were fine-tuned for the lupus nephritis and kidney transplant rejection datasets. The model fine-tuned for lupus nephritis was also applied to the angiomyolipoma sample. Nucleus segmentations were dilated by ∼1  μm (7 pixels) to achieve approximate whole-cell segmentations. Voronoi tessellation was used to avoid overlap of the expanded nuclei in areas of crowded cells.

### 3.6 Cell Classification

**Fig. 3:**
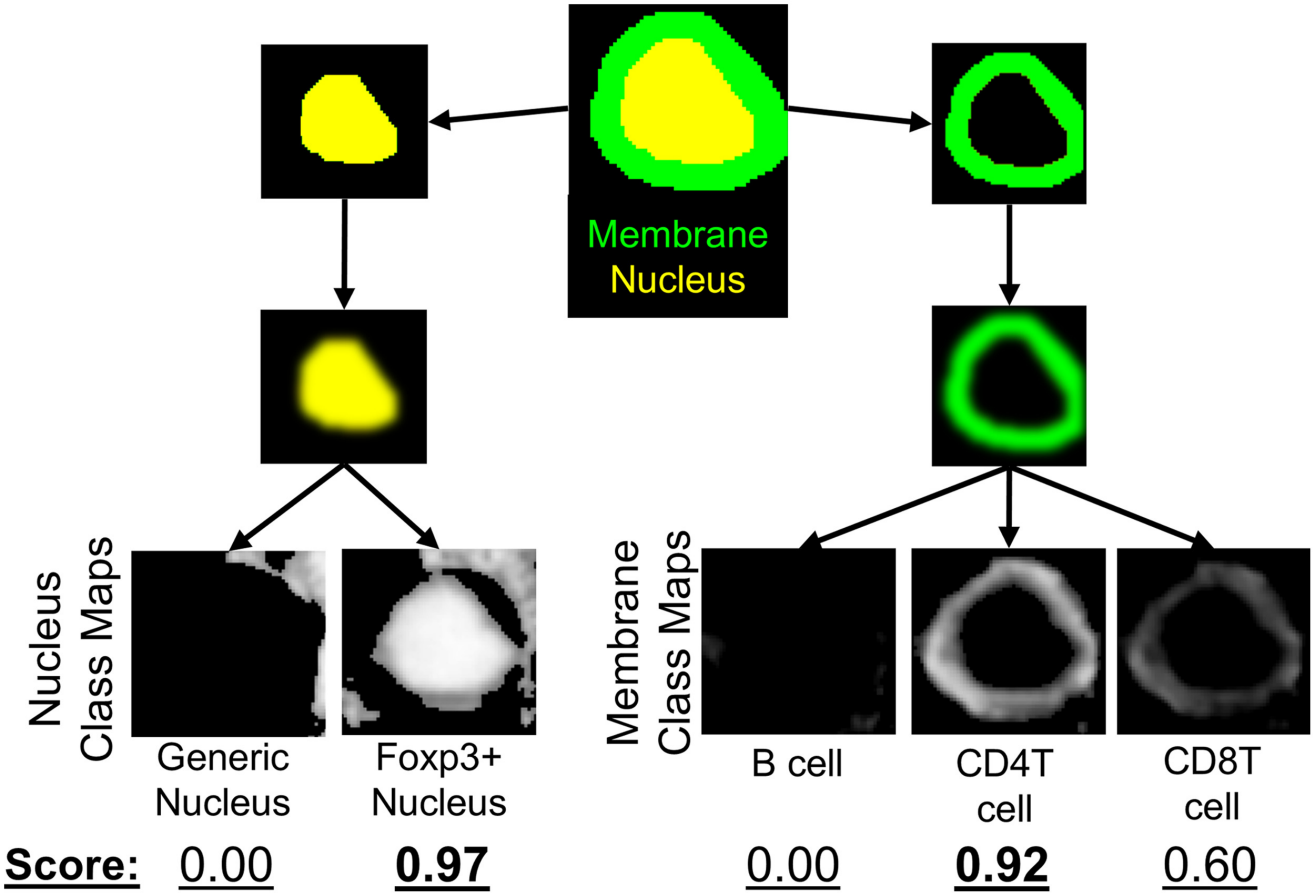
Class maps are combined with instance segmentations of cells to classify cells. The segmented nuclei and a dilated ring around each nucleus are used to mask class maps to generate class scores for each detected cell. The maximum score in each compartment provides a compartment-specific class for every cell.

To optimize our cell classification, a threshold of 0.5 was applied to all class maps to reject low cosine similarity scores. Importantly, this threshold is below the minimum value of the maximum intensity projection (MIP) of all class maps. Cell membrane segmentations were computed by subtracting the nucleus segmentation from the whole-cell segmentation. For each cell predicted by the fine-tuned Cellpose model, we computed a score for all computed class maps using the following protocol (Fig. 3).

A “soft” mask of each nucleus segmentation was created using an inverted distance transform to blur the edges of the binary mask. These “soft masks” were multiplied by all class maps associated with the nucleus compartment. The membrane mask (dilated nucleus segmentation mask—nucleus segmentation mask) was also blurred using a distance transform and multiplied with all class maps associated with the membrane compartment. The mean score of the masked class maps was computed for all reference pseudo-spectra for each cell. Each cell was given a nucleus classification and a membrane classification based on the maximum score for each compartment. A cell received a membrane class of “unclassified” if it scored zero for all membrane classes. If the maximum score for the nucleus compartment was zero, the cell received a nucleus classification of “generic nucleus.”

## 4 Results

### 4.1 pSAM Collapses High-Plex Images to Class Maps

pSAM is a method for computing spatial likelihood maps for cell types probed by the markers used in a custom staining panel. In pSAM, a cosine similarity metric is used to evaluate how close a pixel vector is to a reference vector in an N-dimensional space, where N is the number of channels in an image.

**Fig. 4:**
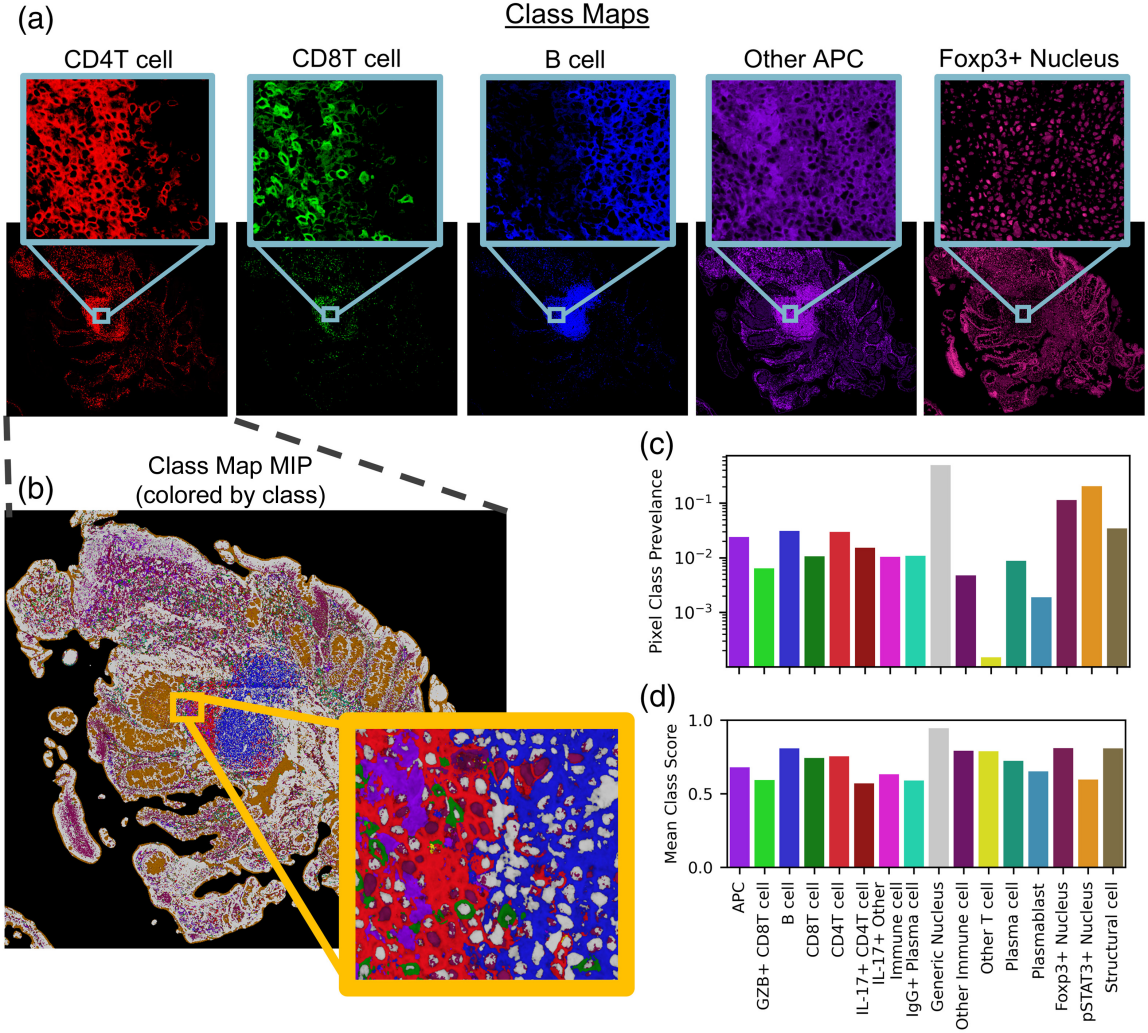
The pSAM class maps display the similarity of pixels to each cell type without the need to compare relative expression across multiple image channels (a). Given the maximum value at each x/y location, individual cells of different populations are clearly visible, even in densely packed areas (b). For the sample shown in panel (b), the prevalence of each pixel class within the tissue was calculated (c). The average cosine similarity score for all classes of pixels in this example image is shown in panel (d). Prevalence (c) and average score (d) are calculated over the pixels that fall within the tissue mask, whereas the slide background is filtered out as background [black in panel (b)].

Class similarity maps for a single reference pseudo-spectrum can compress information from all markers in the panel. Class maps of select reference pseudo-spectra for a full biopsy section are displayed in Fig. 4(a). A maximum intensity projection of all 16 class maps, colored by the argmax [Fig. 4(b)] revealed definitive structures—potentially even tertiary lymphoid structures, and object-level (or cell-level) variability in densely packed areas [Fig. 4(b), inset]. Importantly, the class of the maximum score corresponded to the correct compartments, with nucleus-associated classes scoring highest in cell nuclei, and membrane-associated classes scoring highest in the pixels surrounding cell nuclei. As expected, the highest prevalence pixel class is the “generic nucleus” class, where prevalence is the fraction of the entire population assigned to a class [Fig. 4(c)]. In addition, the “other T cell” class is also very low prevalence, which is expected, as T cell subsets that are negative for both CD4 and CD8 are generally rare. Although very few pixels are most similar to the “other T cell” reference pseudo-spectrum, the average class score (or average cosine similarity across the pixels in this class) for this category was relatively high [Fig. 4(d)]. Conversely, the pSTAT3+ nucleus class showed high pixel prevalence and a relatively low average class score. Within the maximum intensity projection, the areas corresponding to the lumen of the intestine were associated with this class [Fig. 2(b), orange]. The intestinal lumen can be interpreted as background, as there is no tissue in this area.

### 4.2 Pixel Class Maps Aid in Cell Classification

**Fig. 5:**
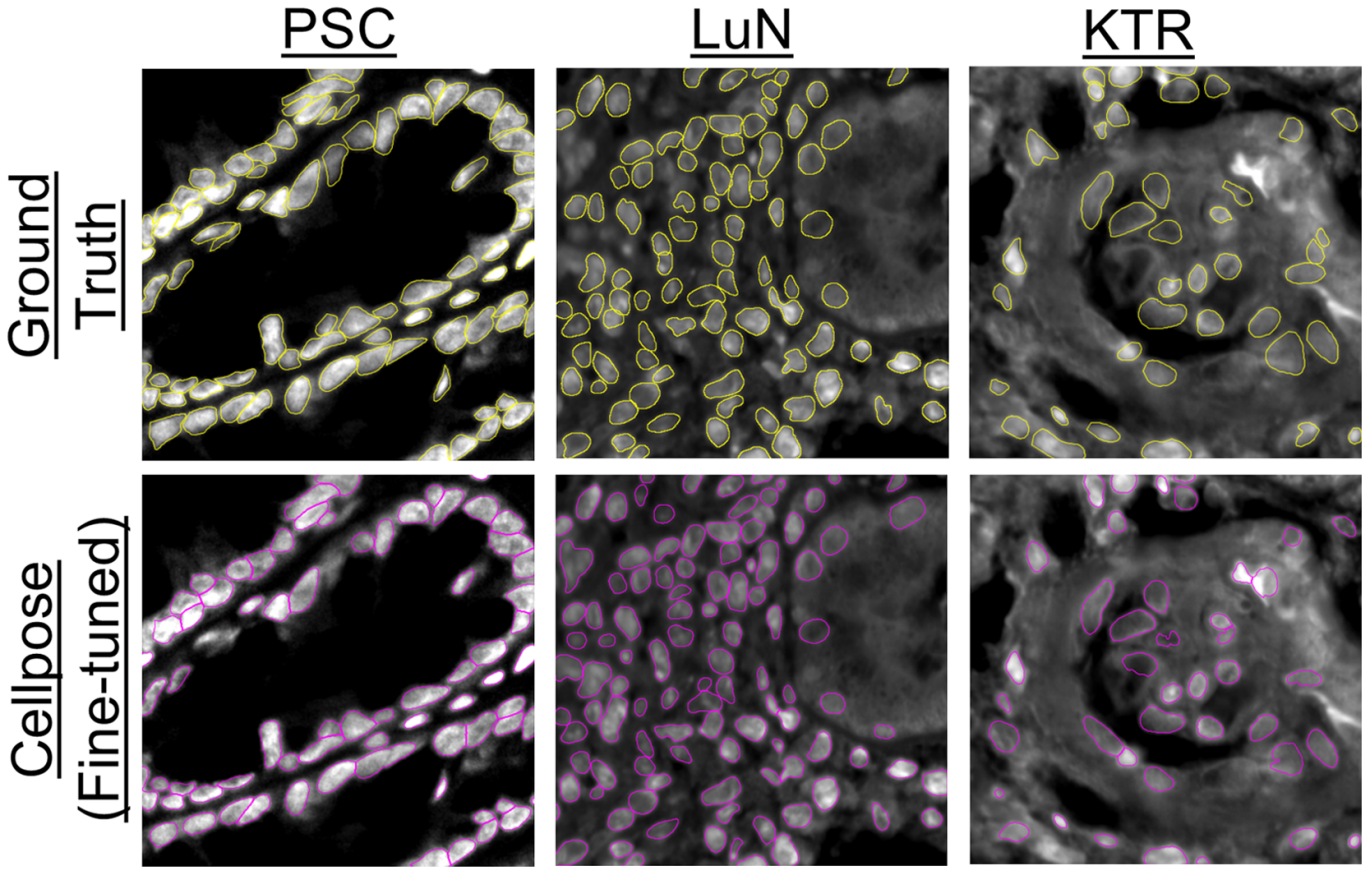
Representative results from three separately fine-tuned Cellpose models on the PSC, LuN, and KTR datasets. Ground truth segmentations (top, yellow) agree well with the fine-tuned models (bottom, magenta) on data from previously unseen patients.

Cellpose2.0 was fine-tuned to detect the cell nuclei in each dataset. After fine-tuning, the performance of each tuned model was evaluated in an independent test set from each dataset (Fig. 5). These test sets comprised 10 non-overlapping image tiles from five separate biopsies for each dataset. Because the angiomyolipoma sample was the only sample from that pathology, the model tuned for the lupus dataset was also used to segment cell nuclei for the angiomyolipoma sample. Note that we demonstrate pSAM in three kidney samples from a larger dataset, so the five samples used for the evaluation of instance segmentation performance are not included in pSAM analysis. In a test set of 50 image tiles per dataset (512×512  pixels), separately fine-tuned models achieved an F1-score of 0.83 ± 0.13 for the PSC dataset, 0.86±0.11 for the LuN dataset, and 0.70±0.16 for the KTR dataset.

**Fig. 6:**
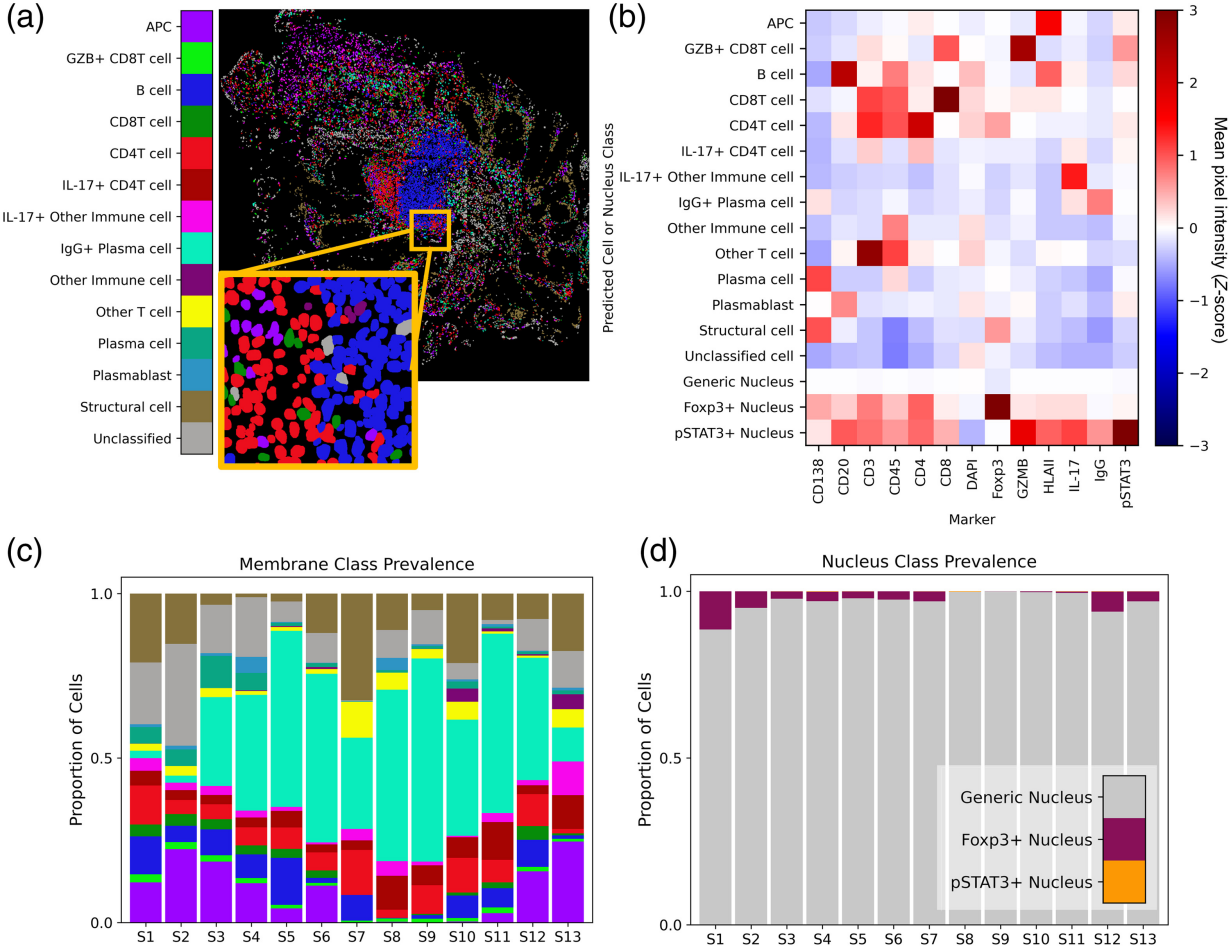
Cell membrane classifications are shown for sample S1 in panel (b). Cell classes were validated using the MPI for all channels for each cell. MPI was Z-scored across the full PSC dataset (13 biopsies, ∼885K cells), and the average Z-score for each channel is calculated for each cell class across all samples (b). Note that each cell received a membrane class (top 13 rows) and a nucleus class (bottom 3 rows). Across the 13 samples in the PSC dataset, pSAM detected varying distributions of cell classes in the membrane compartment (c) and the nucleus compartment (d). Notably, the generic nucleus class (gray) is a strong majority of cells, and pSTAT3+ nuclei (orange) are rare but present and detectable in a few samples. The color bar in panel (a) is also applicable to panel (c).

By collapsing high-plex image data into pixel-level class representations, we were also able to infer likely cell phenotypes—merging pixel-level descriptors with predicted object (cell) masks. Class maps computed using pSAM were combined with instance segmentations of cell nuclei to assign cell classes in the PSC dataset [Fig. 6(a)]. The predicted classes agreed well with the class representations and the fluorescence images themselves [Figs. 2(b) and 6(a)]. These cell phenotype scores were validated by analyzing the MPI for each category of cells across all thirteen colon samples [Fig. 6(b)]. The intensity profiles for each detected cell phenotype corresponded well with the expected relative protein expression for the assigned cell type. This population-level validation demonstrates the potential of pixel-level analysis of highly multiplexed images to improve cell classification. Variation in cell class prevalence was seen across colon samples. Many samples had a plurality of IgG+ plasma cells, whereas others had a much smaller proportion of these cells [Fig. 6(c)]. Across all samples, a strong majority of cells were classified as having a “generic” nucleus [neither Foxp3+ or pSTAT3+, Fig. 6(d)]. Foxp3+ nuclei were much more common than pSTAT3+ nuclei; however, it is worth noting that many samples showed markedly high Foxp3 signal in the villi of the intestine, which may have elevated this prevalence. In agreement with visual inspection of pSTAT3 images, many samples had no pSTAT3+ nuclei, and those that did had a very small proportion. Note that we did not combine membrane and nucleus classes into overall cell classes for this analysis (i.e., regulatory T cells would be CD4T cells or inducible co-stimulator (ICOS)+ CD4T cells with a Foxp3+ nucleus).

### 4.3 Comparing pSAM and Other Cell Classification Methods for High-Plex IF Imaging

Current mainstream methods for classifying cells classification in high-plex IF images draw from single-cell analyses such as flow cytometry and single-cell sequences. Decision trees are generally used in flow cytometry analyses, passing cell populations through “gates” or hand-drawn thresholds at each decision point. In single-cell sequencing, clustering algorithms are generally used to group cells into similar populations. The resulting clusters are manually, iteratively tuned to reach biologically sound classifications. These two methods can also be implemented to classify cells in images, using the MPI of each segmented cell in all channels for gating in a decision tree or as input features into a clustering algorithm.

**Fig. 7:**
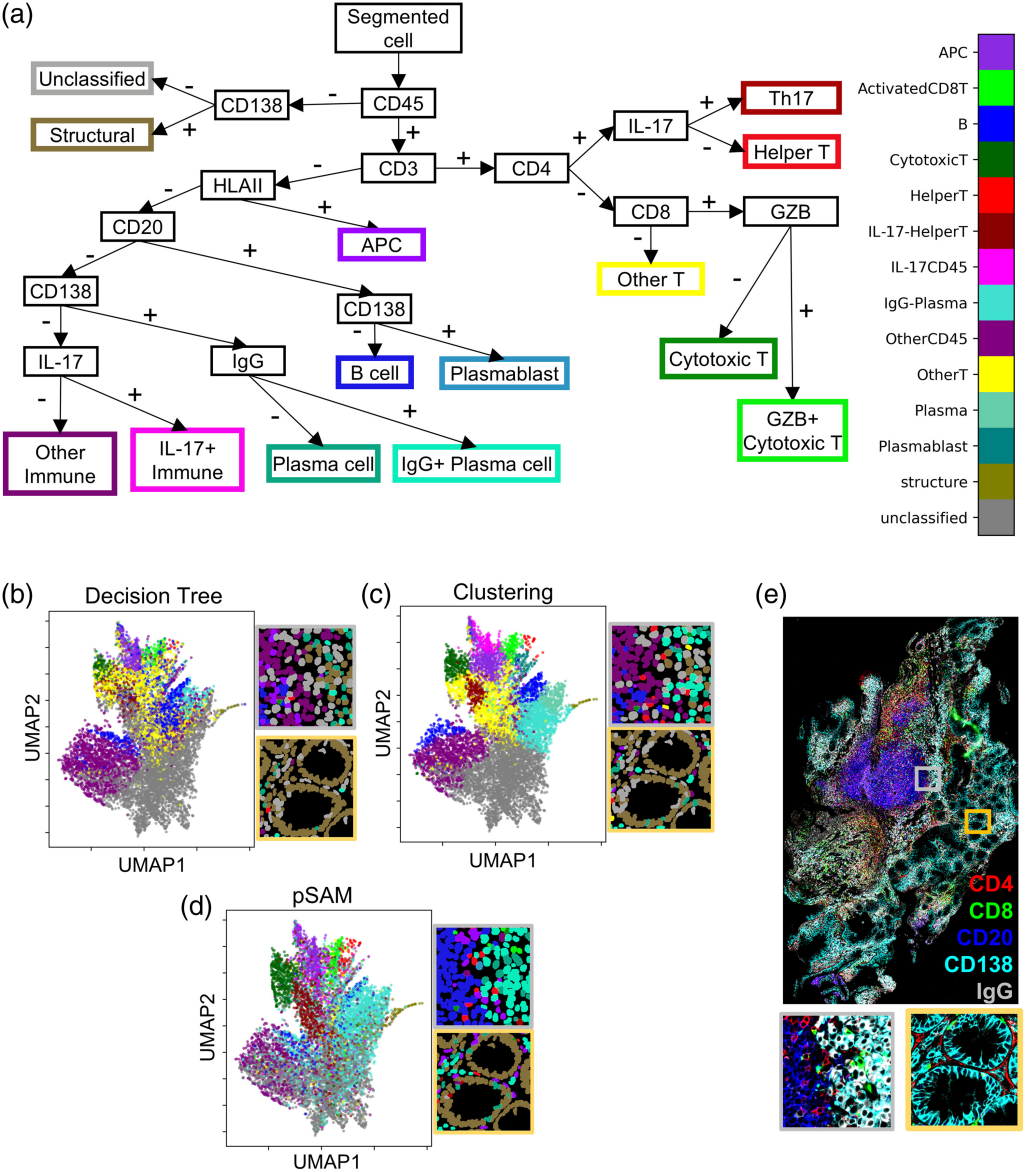
Comparison of pSAM to a decision tree and K-means clustering for cell classification. (a) A decision tree was designed to classify cells in the PSC dataset. Cells are gated at each decision point using a threshold of MPI for the listed marker. UMAP dimensionality reduction was used to compress the 13-dimension feature space into two dimensions, colored by cell class defined by the decision tree (b), K-means clustering (c), and pSAM (d). Cell classes were also mapped back to the global coordinate space [gray and yellow boxes in panels (b)–(d)]. These regions correspond to the gray and yellow insets from the image in panel (e) (selected channels shown).

To compare these methods to pSAM, we have designed a decision tree for classifying cells in the 13-plex PSC data for comparison to the pSAM classification results [Fig. 7(a)].^46^ Thresholds at each decision point were calculated semi-automatically by first implementing a multi-Otsu thresholding then manually selecting one of the calculated thresholds for each decision point in the tree. We also implemented K-means clustering using 11 of the 13 cell markers in the panel. Cell nucleus markers Foxp3 and pSTAT3 were excluded for both the decision tree and clustering to draw direct comparisons to the pSAM “membrane-based” classification.

Uniform manifold approximation and projection (UMAP) dimensionality reduction was used to visualize all 13 MPI features in a two-dimensional feature space, and the resulting plot was colored by the cell classification used by the decision tree [Fig. 7(b)], K-means clustering [Fig. 7(c)], and pSAM [Fig. 7(d)]. Similar areas within the UMAP space correspond to the same cell classes across the three methods. The decision tree and K-means clustering result in a larger group of unclassified cells relative to pSAM. In addition, the “other T cell” class [yellow in Figs. 7(b)–7(d)] was over-represented in K-means clustering, as it should be a rare cell class. In addition, the decision tree showed comparatively fewer plasma cells and IgG+ plasma cells relative to clustering and pSAM. Notably, IgG+ plasma cells have been shown to be an abundant cell type in PSC.^44^

Visualizations of the classified cells from each method [Figs. 7(b)–7(d)] show that pSAM accurately depicts a stark boundary between B cells and plasma cells [Fig. 7(e), gray box], whereas both the decision tree and K-means clustering predict a region of “other immune cells” between the B cell and plasma cell regions. Similarly, pSAM leaves fewer cells unclassified in the villi of the intestine [Figs. 7(b)–7(e), yellow inset].

### 4.4 pSAM is Readily Generalizable to New Imaging Experiments

**Fig. 8:**
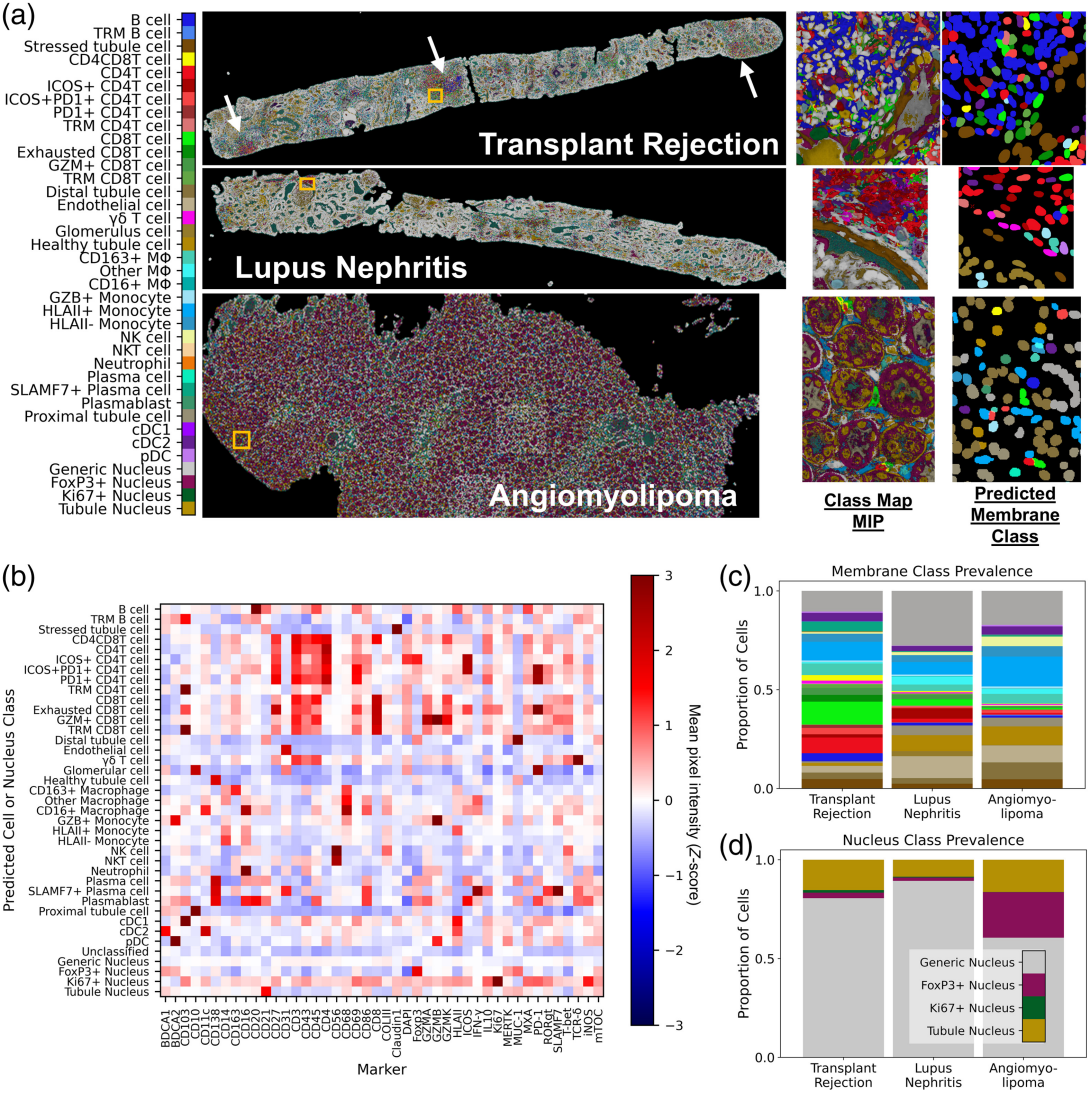
pSAM is readily applied to new datasets. (a) pSAM was used to classify pixels and cells in three kidney samples. Each cell received a class assignment for one of 34 membrane classes and one of four nucleus classes (data not shown). White arrows in the KTR sample denote immune cell aggregates. Insets at the location of the yellow box are shown to the right of the whole-section class map MIPs. Predicted cell classes for the inset are shown at the far right. (b) The Z-score of the population MPI for all stains in the 43-marker panel is compared across all cell membrane and nucleus classes. Note that all cells receive both a membrane class and a nucleus class. (c) Comparison of the relative populations in each sample shows that the immune populations detected show a diverse and abundant population of immune cells, corresponding with visual inspection of the images. (d) Tubule and generic nuclei (gold and gray, respectively) are by far the most abundant nuclei in all three samples. The color bar in panel (a) is also applicable for panel (c).

High-plex IF imaging experiments vary widely in the protein marker panel used to probe cells and tissue. Therefore, to evaluate the generalizability of pSAM, we applied the same method used on the 13-marker PSC dataset to a pilot set of three kidney samples imaged with the same 43-marker staining panel. The higher plex staining panel used in the kidney dataset allowed for more than double the number of cell types and states to be detected in this dataset relative to the 13-plex PSC dataset. Pixels and cells of a given class were consistently identified across all three kidney samples, despite coming from patients with different diagnoses. In addition, the pixel class prevalence trend as expected; nucleus-associated classes had the highest pixel prevalence, and more inflamed samples showed a higher prevalence of pixels with high similarity to immune cell reference pseudo-spectra (Fig. S3 in the Supplementary Material). Specifically, the KTR sample has by far the highest prevalence of lymphocyte-associate pixels (T and B cells), and this sample has three large clusters of lymphocytes visible in the image [Fig. 8(a), white arrows]. Cell classes were also computed using the instance segmentation masks and the pixel class representations.

Validation with MPI across each predicted class of cells showed that each cell phenotype had the expected relative protein expression [Fig. 8(b)]. The trends for protein expression of a given cell type are similar across these samples from different pathological conditions (Fig. S4 in the Supplementary Material). All 43 markers were used for MPI validation, whereas only 30 markers were used for cell classification. Therefore, a few surprising trends were found with the remaining 13 markers. For example, RAR-related orphan receptor-γt (ROR-γt) (not used in cell classification) was relatively high in SLAMF7+ plasma cells. Further investigation is needed to determine whether this finding is associated with cross-reactivity of antibodies or spatial overlap of signal from neighboring cells. Further validation of the pSAM cell classification showed that the prevalence of immune cell populations is highest in the transplant rejection sample, which corresponds with visual analysis of the full biopsy sections [Fig. 8(c)]. In addition, the generic nucleus class is by far the most abundant across samples, as expected [Fig. 8(d)].

### 4.5 Integrating Cell Classification with Interstitial Pixel Classes

**Fig. 9:**
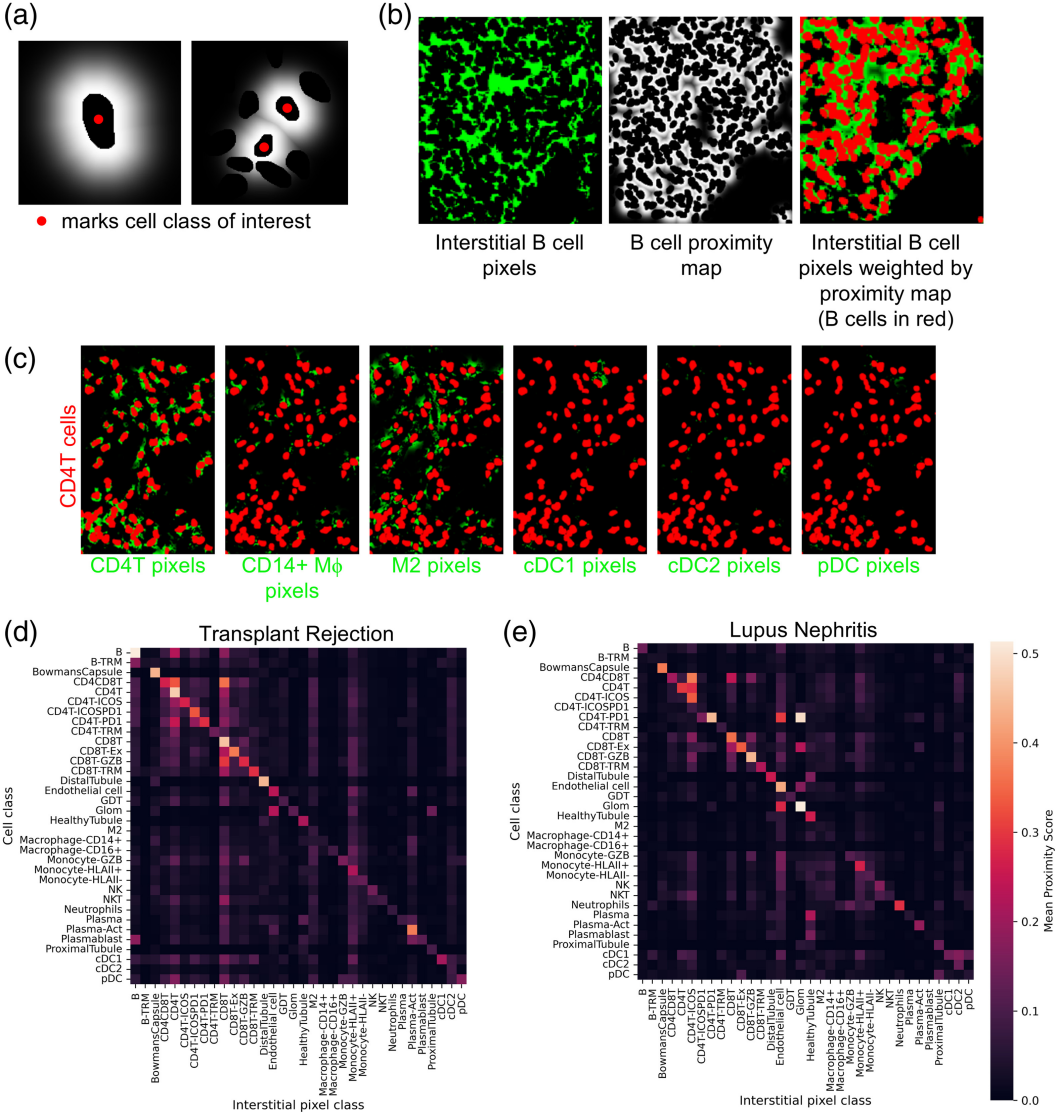
Evaluating interstitial pixels through proximity mapping to cells. (a) For each cell class, proximity maps were calculated by weighting interstitial pixels with a Gaussian decay away from the boundaries of cells from that class. For sparse areas of cells (left), this results in a smooth decay. In areas of crowded cells (right), pixels belonging to cells of other classes are weighted at zero, as they are not “interstitial.” (b) By combining interstitial pixel class maps with the proximity maps from panel (a), each class of interstitial pixels can be scored relative to a given cell class. (c) Six interstitial pixel class maps are combined with the proximity map from CD4T cells to demonstrate the variability in proximity scores for different classes of interstitial pixels. CD4T interstitial pixels are generally located near the CD4T cell boundaries, as nucleus dilation is not a perfect segmentation strategy. Mφ pixels (both CD14+ Mφ and M2) are in close proximity to CD4T cells, suggesting that out-of-plane cells might be interacting CD4T cells. Interstitial pixels from DC classes also show infrequent high proximity scores. All interstitial pixel classes were scored in relation to all cell classes for the kidney transplant rejection sample (c) and the lupus nephritis sample (d).

One of the benefits of pSAM is that both pixel and cell classifications can be calculated. Therefore, the complexity of the interstitial space between cells can also be analyzed. This is particularly important for contextualizing interstitial signal from irregularly shaped myeloid cells, such as macrophages and dendritic cells, as their protrusions are likely present in image planes where their cell nucleus cannot be captured. To demonstrate the utility of pSAM for this type of analysis, we have developed a “proximity score” metric for evaluating whether pixels of a given class preferentially fall near cells of a given class, suggesting that out-of-plane cells might be interacting with cells in the imaging plane. To calculate this proximity score, a proximity map for a given cell class is calculated [Fig. 9(a)]. First, pixels in the interstitial space (pixels that are in the tissue but lie outside of cell segmentations) are weighted with a Gaussian decay with respect to distance from the cell edge [Eq. (3)]. Sigma (σ) was selected to be 22.36 pixels, so that pixels 4  μm from the cell boundary will be weighted at 0.5. If a cell has no neighbors within the vicinity, this results in a smooth decay away from the cell [Fig. 9(a), left]. However, if there are cells from other classes that reside near cells in the class of interest, the pixels within those cell segmentations are weighted at zero, as they are not interstitial pixels [Fig. 9(a), right].

Next, for each class defined by pSAM, a binary mask of pixels belonging to this class was multiplied by the proximity map defined for each cell class [Fig. 9(b)]. For each unique combination of cell and pixel class, the proximity score was calculated as the mean non-zero value. Proximity scores were calculated for the lupus nephritis sample and the kidney transplant rejection sample from above. As expected, the highest proximity scores are for matched cell and pixel classes along the diagonal [Figs. 9(c) and 9(d)]. Other high values indicate that pixels from one class are preferentially near cells of another, suggesting potential interactions. This type of analysis could augment a cell-centric analysis that infers interactions from the proximity of cell centroids. Importantly, this proximity score, facilitated by pSAM, can incorporate contextual information from the image that does not rely solely on detected cells, which can help to infer cell:cell interactions between in-plane and out-of-plane cells.

## 5 Discussion

pSAM provides a new tool for analyzing pixels and cells in highly multiplexed microscopy images. Compressing multi-channel image data into class representations can help facilitate rapid viewing of these complex images and evaluate the cellular constituents of a biopsy sample. In this work, we demonstrated the benefits of pSAM for pixel and cell classification in two different image datasets imaged with very different staining panels. First, in 13-plex images of colon biopsies from PSC/IBD patients, pSAM class maps demonstrated that compressing the channel dimension of these images allows for rapid visualization of unique cell types in dense regions of immune cell infiltrates. Not only were these class maps informative at the pixel and regional level, combining them with an open-source cell-by-cell masking algorithm produced sensible cell classifications.

Manual annotation of cell classes in high-plex images is a particularly difficult task for human readers. Although human readers are the “gold standard” for this task, they are noisy and inconsistent.^21^^,^^47^^,^^48^ Supervised classification algorithms using image annotations are therefore limited by the ambiguity of the ground truth. pSAM provides a means for classifying cells that does not rely on manual image annotations and therefore does not depend on ground truth that is not only noisy and unreliable but also difficult and costly to obtain.

**Fig. 10:**
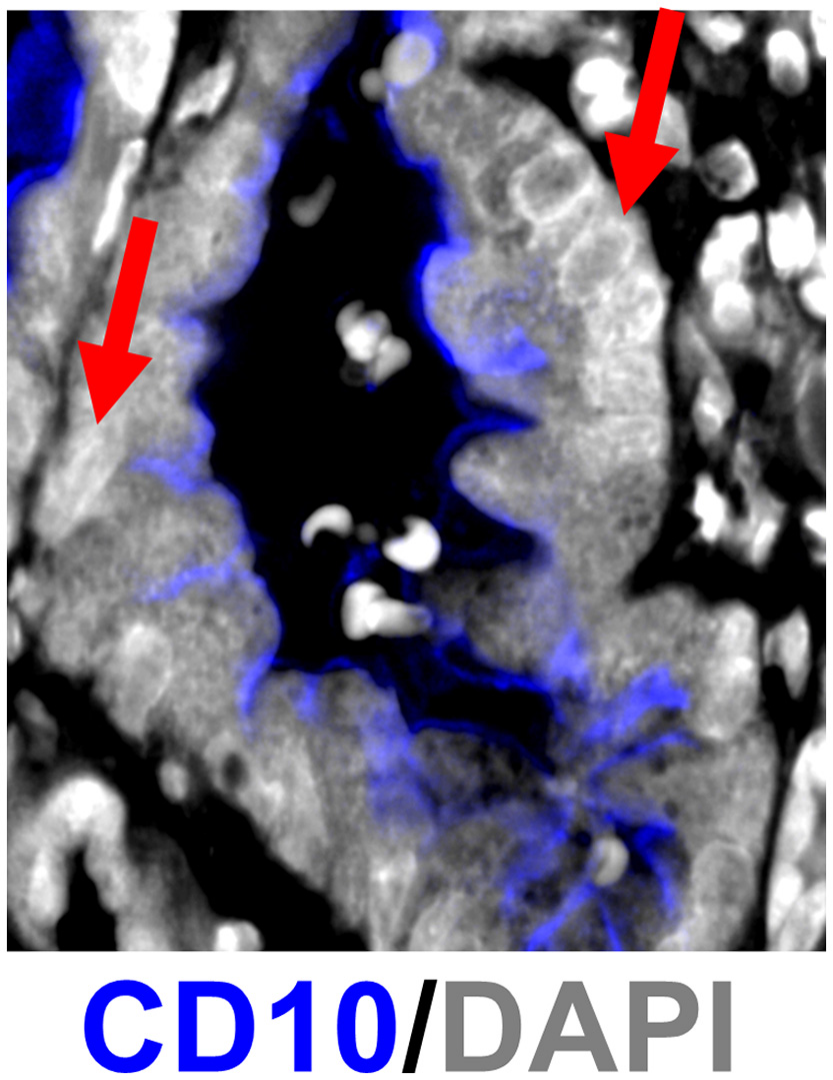
Representative CD10 expression (distal tubule marker) in a kidney sample. CD10 signal resides outside of the range of nucleus dilation for many tubule cells (select tubule cells denoted by red arrows).

In the 43-plex kidney images, pSAM detected a wide range of cell classes with highly variable prevalences, suggesting that it can accurately detect rare cell types. However, there were some discrepancies in the angiomyolipoma sample with tubule cells often classified as monocytes and pDCs. Upon visual inspection of the images, the tubules in this sample are highly irregular and have high levels of expression or cross-reactivity with CD14 (a marker for myeloid cells) and BDCA2 (a marker for pDCs). Foxp3 is also high in the tubule cell cytoplasm in this sample, but because that marker is only high in a nucleus-associated reference, this cross-reactivity does not affect cell classification. In addition, the markers in the staining panel that are intended for tubules stain the brush border of the tubule structure and not the cell membrane or the cytoplasm near the nucleus (Fig. 10).

The localization of the tubule marker signal to the tubule structure and not the individual cells would result in poor cell classification when classifying a cell based on the nucleus dilation methods shown here. Despite this limitation, the class maps show high agreement with the tubule classes at the brush border of the tubule and therefore maintain important contextual information that would be lost in a “detect-first” classification method. Therefore, pSAM provides a rapidly adaptable tool for assessing pixel classes which are helpful for inferring cell type prevalence in images of diverse populations of cells.

High-plex imaging is often used for biological discovery, and the stains used are subject to change from experiment to experiment as researchers toggle the cell types and populations they want to probe. The acquisition of robust and reliable ground truth for every new imaging experiment is costly and time-consuming, even if the quantity of manual annotations is reduced by fine-tuning an existing model. Supervised classification of cells in highly multiplexed images can be particularly difficult because image annotations are both difficult to acquire and specific to the staining panel used for the imaging experiment.^48^ Therefore, models trained for cell classification in high-plex images are not typically generalizable to new datasets.^49^ In addition, the identification of rare cell types is also problematic because they or often not represented in training data. pSAM requires no image annotations—no noisy ground truth from human readers. Although pSAM is a supervised algorithm, all supervision comes from generating reference pseudo-spectra based on the staining panel used in the experiment. Notably, pSAM is less susceptible to batch effects than other methods, such as those inspired by flow cytometry analysis. Because the cosine similarities are calculated within a sample and explicitly evaluating relative intensities, classification is less sensitive to the absolute value of a signal. In addition, although not demonstrated in this work, vector similarity metrics can also be used for spectral unmixing.^33^ In densely packed regions of cells, different cell types can have overlapping signals at the cell membrane.^25^ This overlapping signal problem, or “mixed signal” can also potentially be addressed through spectral angle methods by allowing linear combinations of reference pseudo-spectra.^22^

Although pSAM is an adaptable and readily implemented algorithm for pixel and cell classification, the method is not without its limitations. Pixel-level analyses require pixel-level calculations. Although a cosine similarity function is very quick to calculate, whole-slide images tend to be several giga-pixels in size, and the class maps computed by pSAM have the same dimensions as a single channel of the image. This necessitates a large amount of disk space relative to “detect-first” methods which begin by reducing the data footprint from the number of pixels in an image ($∼10^{8}$) to the number of cells in an image ($∼10^{4}$ to $10^{5}$). Also, many current studies ignore pixels that do not belong to detected cells. These “interstitial pixels” could simply contain noise or nonspecific antibody staining. However, if these pixels have a very high similarity to a reference pseudo-spectrum, it is much less likely to be non-specific staining, as multiple markers would have preferentially stained (and not stained) that location. Maintaining information from these pixels allows for a more thorough analysis of the tissue, contextualizing the environment of the detected cells. Pixel-level analysis is currently an uncommon technique for high-plex image analysis. Liu et al.^1^ have developed another pixel-level analysis method and demonstrated that random sampling of pixel vectors from an image yields reproducible classes of pixels. Although powerful, this sampling could miss rare cell types that could be easily detected with pSAM.

High-plex imaging experiments can also be used to probe unexpected cell types or characterize the interstitial or acellular spaces in tissue.^30^ The pSAM algorithm presented here can only detect the classes defined by the reference pseudo-spectra. In general, staining panels—even high-plex panels—are experimentally designed to probe known cell types or states. Therefore, the “you can only find what you look for” limitation of pSAM and other library-based methods may not be a limitation for many imaging experiments, particularly those in which researchers are interested in the spatial distributions of known or specific cell types. In addition, if there is a plausible, but unexpected cell type, testing the existence of this cell type would only require the generation of a reference pseudo-spectra to match that type.

Finally, cell classification with pSAM class maps is currently optimized to classify cells with small cell bodies—including lymphoid cells, endothelial cells, and some myeloid cells. As mentioned earlier, tubule cells in the kidney are often characterized by signals at the brush border of the tubule, which can be far away from the tubule cell nuclei. A simple nuclear dilation often does not catch the associated cell signal. In this work, we demonstrate that pSAM class maps can aid cell classification by summarizing pixel class scores within a dilated nucleus segmentation to classify the cell. Other cell classification methods are also currently limited by the accuracy of their whole-cell segmentations, as they quantify signal—rather than class score—within the segmentation. More accurate whole-cell segmentations would improve cell classification, regardless of the annotation method used. However, as pSAM also provides pixel annotations, information from the interstitial space between cells is maintained, and can be further analyzed to investigate potential interactions between in-plane cells and amorphous cells whose nuclei reside outside of the imaging plane.

## 6 Conclusion

Overall, pSAM is a simple and elegant solution for classifying pixels and cells in highly multiplexed microscopy data. Although the method has its limitations, it provides a generalizable and easily implementable solution for compressing high-channel image data into lower-dimensional class representations. Importantly, pSAM mitigates the need to collect manual annotations on images or individual cells, whereas probing the cell classes and states that a high-plex panel was designed to detect.

## Supplementary Material



## Data Availability

All relevant code will be available upon publication at https://github.com/durkeems13/pSAM Image data from the PSC dataset will be available through HuBMAP.

## References

[1] Liu C. C. et al., “Robust phenotyping of highly multiplexed tissue imaging data using pixel-level clustering,” Nat. Commun. 14(1), 4618 (2023). NCAOBW 2041-1723 10.1038/s41467-023-40068-5 37528072 PMC10393943

[r2] Greenwald N. F. et al., “Whole-cell segmentation of tissue images with human-level performance using large-scale data annotation and deep learning,” Nat. Biotechnol. 40, 555–565 (2021). NABIF9 1087-0156 10.1038/s41587-021-01094-0 34795433 PMC9010346

[r3] Patwa A. et al., “Multiplexed imaging analysis of the tumor-immune microenvironment reveals predictors of outcome in triple-negative breast cancer,” Commun. Biol. 4(1), 852 (2021). 10.1038/s42003-021-02361-1 34244605 PMC8271023

[r4] Decalf J. Albert M. L. Ziai J., “New tools for pathology: a user’s review of a highly multiplexed method for in situ analysis of protein and RNA expression in tissue,” J. Pathol. 247(5), 650–661 (2019). 10.1002/path.5223 30570141

[r5] Keren L. et al., “A structured tumor-immune microenvironment in triple negative breast cancer revealed by multiplexed ion beam imaging,” Cell 174(6), 1373–1387.e19 (2018). CELLB5 0092-8674 10.1016/j.cell.2018.08.039 30193111 PMC6132072

[r6] Goltsev Y. et al., “Deep profiling of mouse splenic architecture with CODEX multiplexed imaging,” Cell 174(4), 968–981.e15 (2018). CELLB5 0092-8674 10.1016/j.cell.2018.07.010 30078711 PMC6086938

[r7] Schürch C. M. et al., “Coordinated cellular neighborhoods orchestrate antitumoral immunity at the colorectal cancer invasive front,” Cell 182(5), 1341–1359.e19 (2020). CELLB5 0092-8674 10.1016/j.cell.2020.07.005 32763154 PMC7479520

[r8] Lewis S. M. et al., “Spatial omics and multiplexed imaging to explore cancer biology,” Nat. Methods 18(9), 997–1012 (2021).1548-7091 10.1038/s41592-021-01203-6 34341583

[r9] Abraham R. et al., “Specific in situ inflammatory states associate with progression to renal failure in lupus nephritis,” J. Clin. Investig. 132(13), e155350 (2022). 10.1172/JCI155350 35608910 PMC9246394

[10] Stewart R. L. et al., “Spatially-resolved quantification of proteins in triple negative breast cancers reveals differences in the immune microenvironment associated with prognosis,” Sci. Rep. 10(1), 6598 (2020). SRCEC3 2045-2322 10.1038/s41598-020-63539-x 32313087 PMC7170957

[11] Hoover P. et al., “Accelerating medicines partnership: organizational structure and preliminary data from the phase 1 studies of lupus nephritis,” Arthritis Care Res. 72, 233–242 (in eng) (2020). 10.1002/acr.24066 PMC6992476 31502417

[r12] Rao D. A. et al., “Design and application of single-cell RNA sequencing to study kidney immune cells in lupus nephritis,” Nat. Rev. Nephrol. 16(4), 238–250 (2020). 10.1038/s41581-019-0232-6 31853010 PMC7251304

[13] Wu H. et al., “Single-cell transcriptomics of a human kidney allograft biopsy specimen defines a diverse inflammatory response,” J. Amer. Soc. Nephrol. 29(8), 2069 (2018). JASNEU 1046-6673 10.1681/ASN.2018020125 29980650 PMC6065085

[14] Phillips D. et al., “Highly multiplexed phenotyping of immunoregulatory proteins in the tumor microenvironment by CODEX tissue imaging,” Front. Immunol. 12, 687673 (2021). 10.3389/fimmu.2021.687673 34093591 PMC8170307

[15] Hickey J. W. et al., “Strategies for accurate cell type identification in CODEX multiplexed imaging data,” Front. Immunol. 12, 727626 (2021). 10.3389/fimmu.2021.727626 34484237 PMC8415085

[r16] Stoltzfus C. R. et al., “CytoMAP: a spatial analysis toolbox reveals features of myeloid cell organization in lymphoid tissues,” Cell Rep. 31(3), 107523 (2020) (in eng). 10.1016/j.celrep.2020.107523 32320656 PMC7233132

[17] Hickey J. W. et al., “Organization of the human intestine at single-cell resolution,” Nature 619(7970), 572–584 (2023). 10.1038/s41586-023-05915-x 37468586 PMC10356619

[18] Durkee M. S. et al., “Quantifying the effects of biopsy fixation and staining panel design on automatic instance segmentation of immune cells in human lupus nephritis,” J. Biomed. Opt. 26(2), 022910 (2021). JBOPFO 1083-3668 10.1117/1.JBO.26.2.022910 33420765 PMC7791891

[19] Liarski V. M. et al., “Quantifying in situ adaptive immune cell cognate interactions in humans,” Nat. Immunol. 20(4), 503–513 (2019). NRIABX 1474-1733 10.1038/s41590-019-0315-3 30778242 PMC6474677

[20] Durkee M. S. et al., “Deep learning to detect lymphocytes with high phenotypic resolution in highly multiplexed fluorescence microscopy images of triple-negative breast cancer biopsies,” Proc. SPIE 11964, 1196406 (2022). PSISDG 0277-786X 10.1117/12.2610260

[21] Durkee M. S. et al., “Convolutional neural networks detect cells in densely packed images at performance levels similar to human readers,” Proc. SPIE 12467, 124670J (2023). PSISDG 0277-786X 10.1117/12.2654521

[22] Chang C. I., “Hyperspectral target detection: hypothesis testing, signal-to-noise ratio, and spectral angle theories,” IEEE Trans. Geosci. Remote Sens. 60, 1–23 (2022). IGRSD2 0196-2892 10.1109/TGRS.2021.3069716

[r23] Sohn Y. Rebello N. S., “Supervised and unsupervised spectral angle classifiers,” Photogramm. Eng. Remote Sens. 68(12), 1271–1282 (2002).

[24] Kruse F. A. et al., “The spectral image processing system (SIPS)—interactive visualization and analysis of imaging spectrometer data,” Remote Sens. Environ. 44(2), 145–163 (1993). 10.1016/0034-4257(93)90013-N

[25] Bai Y. et al., “Adjacent cell marker lateral spillover compensation and reinforcement for multiplexed images,” Front. Immunol. 12, 652631 (2021). 10.3389/fimmu.2021.652631 34295327 PMC8289709

[26] Chang Y. H. et al., “RESTORE: robust intensity normalization method for multiplexed imaging,” Commun. Biol. 3(1), 111 (2020). 10.1038/s42003-020-0828-1 32152447 PMC7062831

[27] Lee M. Y. et al., “CellSeg: a robust, pre-trained nucleus segmentation and pixel quantification software for highly multiplexed fluorescence images,” BMC Bioinf. 23(1), 46 (2022). BBMIC4 1471-2105 10.1186/s12859-022-04570-9 PMC8767664 35042474

[28] Greenbaum S. et al., “A spatially resolved timeline of the human maternal–fetal interface,” Nature 619(7970), 595–605 (2023). 10.1038/s41586-023-06298-9 37468587 PMC10356615

[29] Li R.-H. Belford G. G., “Instability of decision tree classification algorithms,” in KDD ′02: Proc. Eighth ACM SIGKDD Int. Conf. Knowl. Discov. and Data Mining, pp. 570–575 (2002). 10.1145/775047.775131

[30] Brbić M. et al., “Annotation of spatially resolved single-cell data with STELLAR,” Nat. Methods 19(11), 1411–1418 (2022).1548-7091 10.1038/s41592-022-01651-8 36280720 PMC12186200

[31] Goetz A. F. H., “Three decades of hyperspectral remote sensing of the Earth: a personal view,” Remote Sens. Environ. 113, S5–S16 (2009). 10.1016/j.rse.2007.12.014

[32] Ningliang L. et al., “Gastric cancer diagnosis using hyperspectral imaging with principal component analysis and spectral angle mapper,” J. Biomed. Opt. 25(6), 066005 (2020). JBOPFO 1083-3668 10.1117/1.JBO.25.6.066005 32594664 PMC7320226

[r33] Rabah B. Z. et al., “A new method to change illumination effect reduction based on spectral angle constraint for hyperspectral image unmixing,” IEEE Geosci. Remote Sens. Lett. 8(6), 1110–1114 (2011). 10.1109/LGRS.2011.2157890

[r34] Calin M. A. Parasca S. V. Sr. Manea D., “Comparison of spectral angle mapper and support vector machine classification methods for mapping skin burn using hyperspectral imaging,” Proc. SPIE 10677, 106773P (2018). PSISDG 0277-786X 10.1117/12.2319267

[35] Fei B., “Chapter 3.6—Hyperspectral imaging in medical applications,” in Data Handling in Science and Technology, Amigo J. M. Ed., Vol. 32, pp. 523–565, Elsevier (2019).

[36] Black S. et al., “CODEX multiplexed tissue imaging with DNA-conjugated antibodies,” Nat. Protoc. 16(8), 3802–3835 (2021).1754-2189 10.1038/s41596-021-00556-8 34215862 PMC8647621

[37] Muhlich J. et al., “Stitching and registering highly multiplexed whole slide images of tissues and tumors using ASHLAR software,” bioRxiv 2021.04.20.440625 (2021). 10.1093/bioinformatics/btac544 PMC9525007 35972352

[38] Nicholson L. B., “The immune system,” Essays Biochem. 60(3), 275–301 (2016). 10.1042/EBC20160017 27784777 PMC5091071

[r39] Shipkova M. Wieland E., “Surface markers of lymphocyte activation and markers of cell proliferation,” Clin. Chim. Acta 413(17), 1338–1349 (2012). CCATAR 0009-8981 10.1016/j.cca.2011.11.006 22120733

[r40] Catakovic K. et al., “T cell exhaustion: from pathophysiological basics to tumor immunotherapy,” Cell Commun. Signal. 15(1), 1 (2017). 10.1186/s12964-016-0160-z 28073373 PMC5225559

[41] I. R&D Systems, “Cell marker interactive resource tool,” https://www.rndsystems.com/resources/cell-markers/immune-cells.

[42] Maccari M. E. et al., “A distinct CD38+CD45RA+ population of CD4+, CD8+, and double-negative T cells is controlled by FAS,” J. Exp. Med. 218(2), e20192191 (2020). JEMEAV 0022-1007 10.1084/jem.20192191 PMC7658692 33170215

[r43] Saltz J. et al., “Spatial organization and molecular correlation of tumor-infiltrating lymphocytes using deep learning on pathology images,” Cell Rep. 23(1), 181–193.e7 (2018). 10.1016/j.celrep.2018.03.086 29617659 PMC5943714

[44] Shaw D. G. et al., “Antigen-driven colonic inflammation is associated with development of dysplasia in primary sclerosing cholangitis,” Nat. Med. 29(6), 1520–1529 (2023).1078-8956 10.1038/s41591-023-02372-x 37322120 PMC10287559

[45] Stringer C. Pachitariu M., “Cellpose 2.0: how to train your own model,” bioRxiv, 2022.04.01.486764 (2022). 10.1038/s41592-022-01663-4 PMC9718665 36344832

[46] Madeleine S. D. et al., “Pseudo-spectral angle mapping to improve immune cell classification in highly multiplexed fluorescence microscopy images,” Proc. SPIE 12846, 1284603 (2024). PSISDG 0277-786X 10.1117/12.3003486

[47] Aeffner F. et al., “The gold standard paradox in digital image analysis: manual versus automated scoring as ground truth,” Archiv. Pathol. Lab. Med. 141(9), 1267–1275 (2017). APLMAS 0003-9985 10.5858/arpa.2016-0386-RA 28557614

[48] Durkee M. S. et al., “Generalizations of the Jaccard index and Sørensen index for assessing agreement across multiple readers in object detection and instance segmentation in biomedical imaging,” J. Med. Imaging 10(6), 065503 (2023). JMEIET 0920-5497 10.1117/1.JMI.10.6.065503 PMC13233040 42245921

[49] Amitay Y. et al., “CellSighter: a neural network to classify cells in highly multiplexed images,” Nat. Commun. 14(1), 4302 (2023). NCAOBW 2041-1723 10.1038/s41467-023-40066-7 37463931 PMC10354029

