# Supplementary material for: Pseudo-spectral angle mapping for pixel and cell classification in highly multiplexed immunofluorescence images
Source: J Med Imaging (Bellingham). 2024 Dec 10;11(6):067502. doi: 10.1117/1.JMI.11.6.067502 (PMC11629784; doi:10.1117/1.JMI.11.6.067502)
Supplement: Supplementary file 1 [file JMI_011_067502_SD001.pdf]

Supplementary information for: Pseudo-spectral angle mapping for pixel and cell classification in highly multiplexed immunofluorescence images

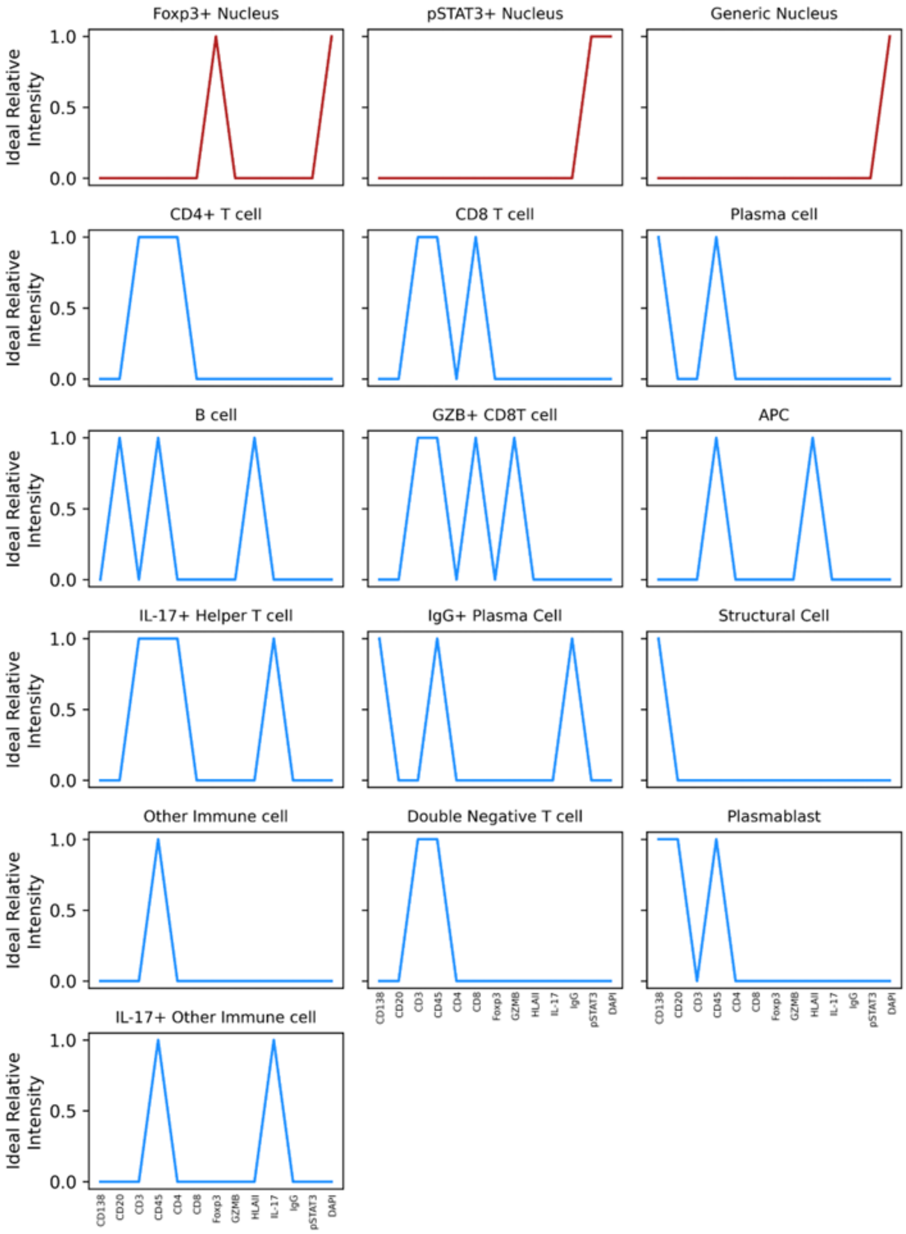

Supplementary Figure 1. All reference pseudospectra are displayed for the thirteen-marker panel used to image the PSC/IBD dataset. Pseudospectra designed to be high in the cell nucleus are displayed in red, while pseudospectra designed to be high in the cell membrane are displayed in blue.

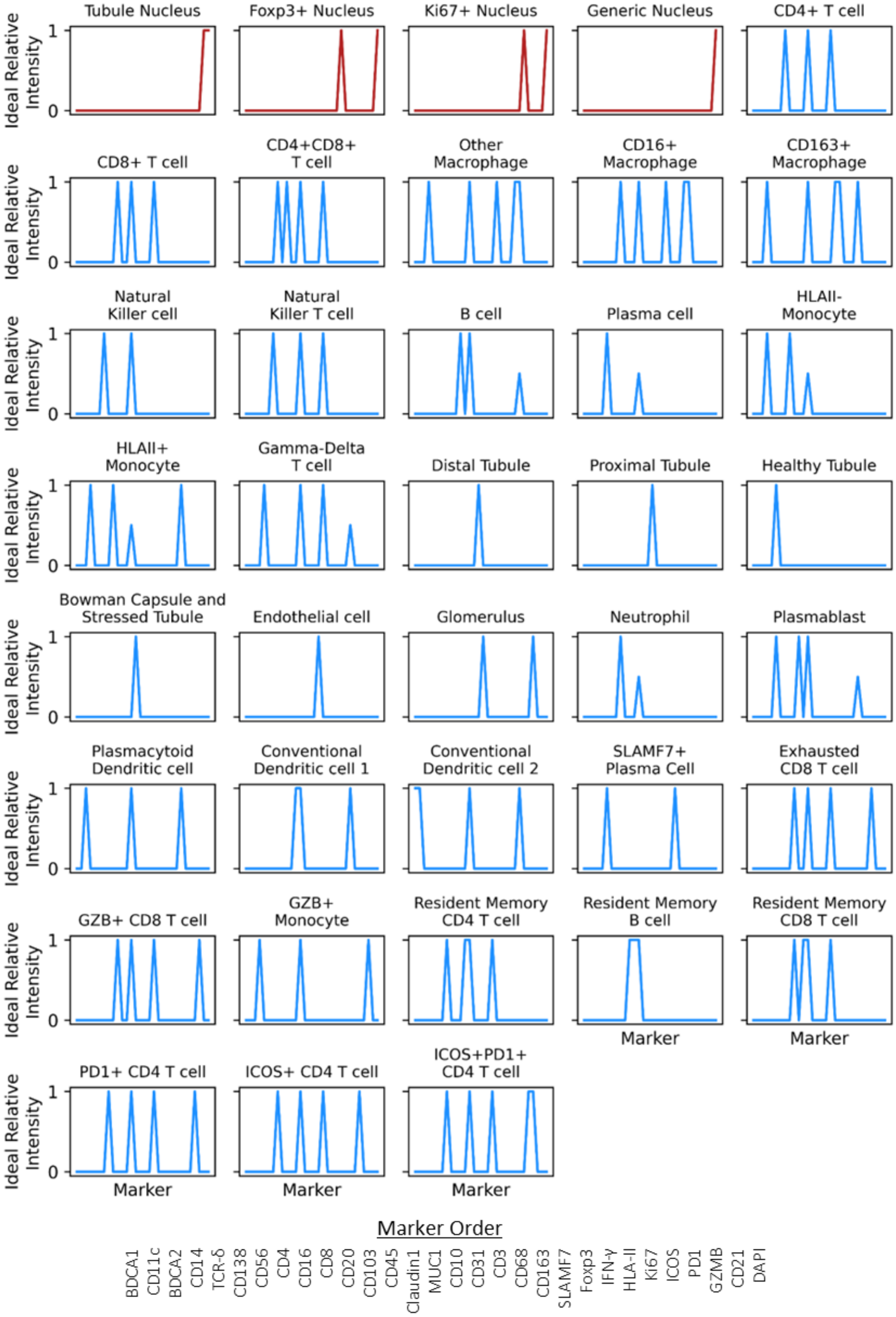

Supplementary Figure 2. All reference pseudospectra are displayed for the 30-marker panel used to image the kidney dataset. Pseudospectra designed to be high in the cell nucleus are displayed in red, while pseudospectra designed to be high in the cell membrane are displayed in blue. The marker order (held consistent across all x-axes in this figure), is displayed at the bottom of the figure.

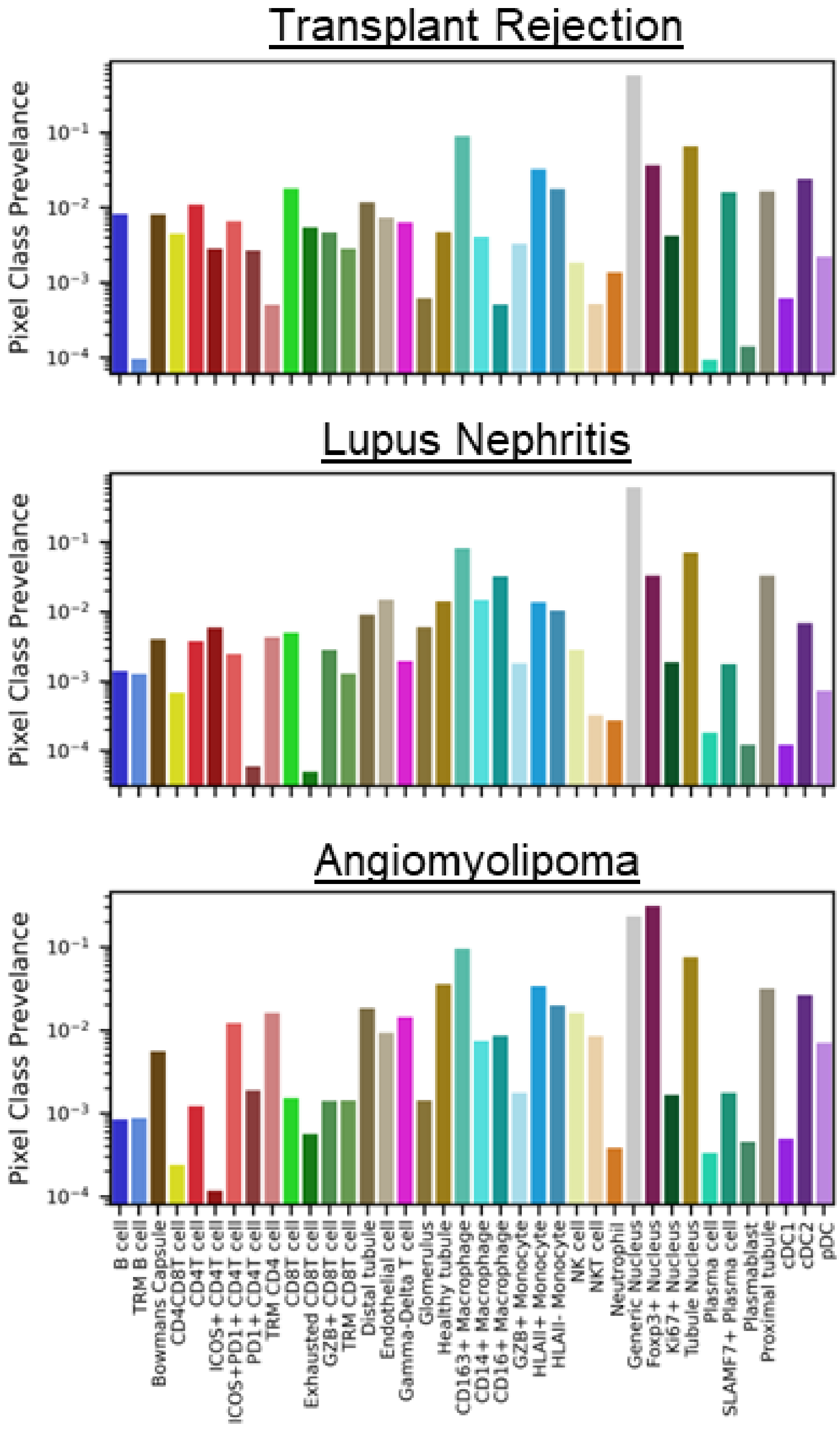

Supplementary Figure 3. Pixel class prevalence (log scale) for all classes is displayed for all three kidney biopsies: transplant rejection (top), lupus nephritis (middle), and angiomyolipoma (bottom).

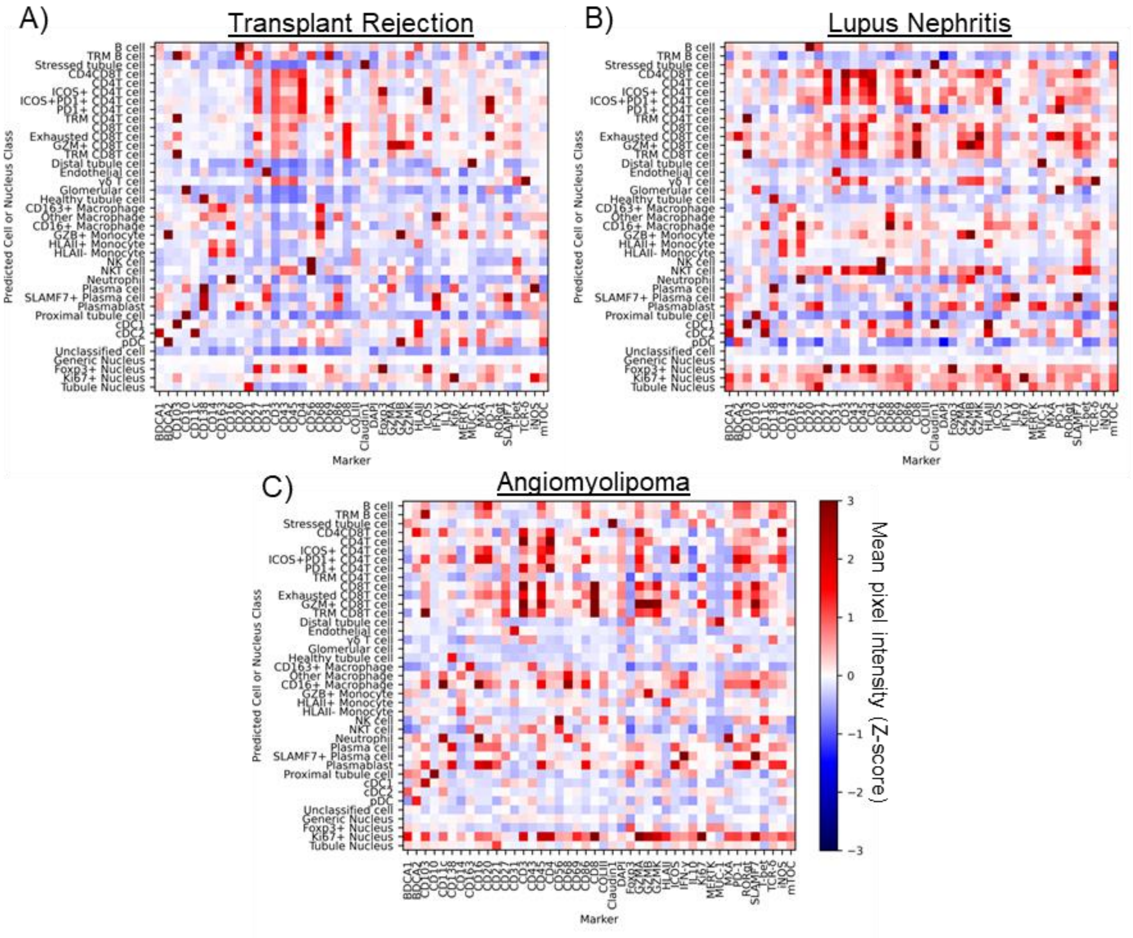

Supplementary Figure 4. Mean pixel intensity (MPI) for all channels in the 43-marker kidney panel by cell class for kidney transplant rejection (A), lupus nephritis (B), and angiolipoma (C). MPIs were z-scored across each channel for each sample.

Marker	Target	Vendor	Cat. No.
CD3	Pan T cell marker	abcam	ab205228
Foxp3	Treg nuclei	eBioscience	14-4776-82
HLAII	Antigen presentation	Abcam	ab7856
CD4	Helper T cells	abcam	ab181724
CD20	B cells	abcam	ab236434
IL-17	TH17 cells	Novus Biologicals	AF-317-NA
CD8	Cytotoxic T cells	Invitrogen	MA5-13473
CD138	Plasma cells/Villi	ThermoFisher Scientific	MA1-10091
pSTAT3	Carcinogenic/Dysplastic cells	abcam	ab219593
GZMB	Activated CD8 T cells	cell signaling	79003SF
IgG	Activated Plasma Cells	abcam	ab226069

Supplementary Table 1. All antibodies used in the PSC/IBD staining panel, including the expected expression for each marker.

Marker	Target	Vendor	Cat. No.
CD31	Endothelial cells	abcam	ab226157
T-bet	TH1 nuclei	Cell signaling	27112SF
CD103	Resident memory cells/cDCs	abcam	ab254201-1001
MUC-1	Distal tubules	Novus Biologicals	NBP2-44658
Foxp3	Treg nuclei	eBioscience	14-4776-82
BDCA1	cDCs	Novus Biologicals	NBP2-70345
CD20	B cells	Invitrogen	14-0202-82
CD10	Proximal Tubules	Abcam	ab256494
MXA	Interferon Response	R&D	AF7946
GZMB	Activated CD8 T cells	cell signaling	79003SF
CD68	Macrophages	eBioscience	14-0688-82
BDCA2	pDCs	Novus Biologicals	AF1376
CD27	B memory cells	abcam	ab272072
GZMA	Activated CD8 T cells	abcam	ab251499
PD-1	CD8 T exhaustion and TFH cells	Cell signaling	63815SF
CD86	T cell activation	Cell signaling	76755
CD45	Pan-immune cell marker	Biolegend	11-9459-42
ICOS	TFH cells	Cell signaling	39740SF
GZMK	Activated CD8 T cells	abcam	EPR24601-164
CD4	Helper T cells	abcam	ab181724
RORγt	TH17 nuclei	ThermoFisher Scientific	PA534164
mTOC	Cell:cell interaction (with CD43)	abcam	ab27074
CD43	Cell:cell interaction (with mTOC)	Novus Biologicals	NBP2-34775
CD69	Lymphocyte activation	abcam	ab234512
IFN-γ	Macrophages	abcam	ab231301
CD163	M2 macrophages	Novus Biologicals	NBP110-40686
CD21	Germinal center B cells*	abcam	ab271855
HLAII-DR	Antigen presentation	abcam	ab7856
CD8	Cytotoxic T cells	Invitrogen	MA5-13473
CD16	Neutrophils, NKs, Monocytes, Macrophages	Cellsignaling	72204SF
TCR-δ	γδ T cells	Santa Cruz	sc-100289
CD138	Plasma cells	ThermoFisher Scientific	MA1-10091
CD11c	cDCs	abcam	EP1347Y
CD3	Pan T cell marker	abcam	ab271850
IL-10	TH1/TH2 cells	Novus Biologicals	AF-217-NA
Ki67	Cell proliferation	abcam	ab279657
Claudin1	Bowman's Capsule**	abcam	EPR121871
COLIII	Collagen	Proteintech	22734-1-AP
MerTK	Phagocytosis	abcam	ab271851
iNOS	Inflammatory macrophages	abcam	ab239990
CD14	Monocytes, macrophages	abcam	ab230903-1001
SLAMF7	Activated Plasma cells	Cell signaling	98611S
CD56	NK and NKT cells	OriGene	CF506208

Supplementary Table 2. All antibodies used in the kidney staining panel, including the expected expression for each marker.
